# MicroRNA 483‐3p overexpression unleashes invasive growth of metastatic colorectal cancer via *NDRG1* downregulation and ensuing activation of the ERBB3/AKT axis

**DOI:** 10.1002/1878-0261.13408

**Published:** 2023-03-19

**Authors:** Ermes Candiello, Gigliola Reato, Federica Verginelli, Gennaro Gambardella, Antonio D′Ambrosio, Noemi Calandra, Francesca Orzan, Antonella Iuliano, Raffaella Albano, Francesco Sassi, Paolo Luraghi, Paolo M. Comoglio, Andrea Bertotti, Livio Trusolino, Carla Boccaccio

**Affiliations:** ^1^ Laboratory of Cancer Stem Cell Research Candiolo Cancer Institute, FPO‐IRCCS Turin Italy; ^2^ Department of Oncology University of Turin Medical School Italy; ^3^ Telethon Institute of Genetics and Medicine (TIGEM) Naples Italy; ^4^ Department of Chemical Materials and Industrial Engineering University of Naples Federico II Italy; ^5^ Translational Cancer Medicine Candiolo Cancer Institute, FPO‐IRCCS Turin Italy; ^6^ IFOM, FIRC Institute of Molecular Oncology Milan Italy

**Keywords:** cancer stem cell, colorectal cancer, ERBB3, metastasis, miRNA‐483‐3p, NDRG1

## Abstract

In colorectal cancer, the mechanisms underlying tumor aggressiveness require further elucidation. Taking advantage of a large panel of human metastatic colorectal cancer xenografts and matched stem‐like cell cultures (m‐colospheres), here we show that the overexpression of microRNA 483‐3p (miRNA‐483‐3p; also known as MIR‐483‐3p), encoded by a frequently amplified gene locus, confers an aggressive phenotype. In m‐colospheres, endogenous or ectopic miRNA‐483‐3p overexpression increased proliferative response, invasiveness, stem cell frequency, and resistance to differentiation. Transcriptomic analyses and functional validation found that miRNA‐483‐3p directly targets NDRG1, known as a metastasis suppressor involved in EGFR family downregulation. Mechanistically, miRNA‐483‐3p overexpression induced the signaling pathway triggered by ERBB3, including AKT and GSK3β, and led to the activation of transcription factors regulating epithelial–mesenchymal transition (EMT). Consistently, treatment with selective anti‐ERBB3 antibodies counteracted the invasive growth of miRNA‐483‐3p‐overexpressing m‐colospheres. In human colorectal tumors, miRNA‐483‐3p expression inversely correlated with NDRG1 and directly correlated with EMT transcription factor expression and poor prognosis. These results unveil a previously unrecognized link between miRNA‐483‐3p, NDRG1, and ERBB3‐AKT signaling that can directly support colorectal cancer invasion and is amenable to therapeutic targeting.

## Graphical abstract

MicroRNA‐483‐3p (miRNA‐483‐3p) is a putative oncogene playing a still elusive role in colorectal cancer progression. We show that miRNA‐483‐3p directly targets NDRG1, known as a ‘metastasis suppressor’. Following NDRG1 downregulation, EGFR and ERBB3 signaling are unleashed, promoting retention of stemness features, epithelial–mesenchymal transition, and invasive growth. These properties can be counteracted by ERBB3 inhibition, offering a chance for therapeutic intervention.

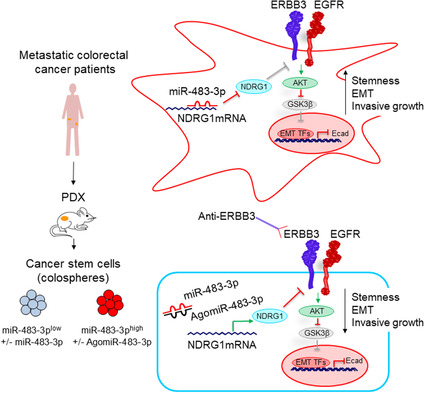

**Abbreviations**
CCLE: Cancer Cell Line Encyclopedia
CRC: colorectal cancer
EGF: epidermal growth factor
EGFR: epidermal growth factor receptor
EMT: epithelial–mesenchymal transition
FGF2: fibroblast growth factor 2
IGF2: insulin‐like growth factor 2
LDA: limiting dilution assay
NDRG1: N‐myc downstream‐regulated gene
PDX: patient‐derived xenograft
PLA: proximity ligation assay
TCGA: The Cancer Genome Atlas

## 1 Introduction

Metastatic colorectal cancer (CRC) is the third most common cause of cancer‐associated death worldwide, with 5‐year survival rates < 15% [1, 2]. Increasing evidence suggests that cancer stem‐like cells have a central role in recurrence after both conventional and targeted therapy and in metastatic dissemination of many cancer types, including CRC [3, 4].

From a cohort of metastatic CRC propagated as patient‐derived xenografts (PDX or xenopatients) [5], we previously derived stem‐like cells that were long‐term propagated as cultures hereafter named m‐colospheres [6, 7]. These cells faithfully retained the genetic traits of the original tumors, together with the genetically driven response to targeted therapy [6, 7]. A subset of xenopatients and m‐colospheres without genetic alterations of *KRAS*, *NRAS*, *PIK3CA*, or *BRAF*, henceforth defined as ‘quadruple wild‐type (WT)’, and including the majority of cases, were found to be strongly relying on epidermal growth factor receptor (EGFR) and MET signaling, and thus sensitive to targeted inhibition of these receptors [5, 6, 7]. However, within this tumor subset, a subgroup of xenopatients and m‐colospheres displayed proliferative ability independent of EGFR (resistance to EGFR inhibition), associated with overexpression and actionability of insulin‐like growth factor 2 (IGF2) [7, 8].

Interestingly, the *IGF2* locus, located on chromosome 11p15.5 and amplified in 7% of CRCs, encodes also miRNA‐483 [9, 10]. In CRCs harboring an amplified locus, both *IGF2* and miRNA‐483 were found overexpressed and miRNA‐483 was validated in organoids as the dominant driver oncogene [9, 11]. More recently, the importance of the IGF2/miRNA‐483 locus in CRC was confirmed by the evidence that the locus undergoes ‘enhancer hijacking’, by formation of an aberrant contact domain with a lineage‐specific super‐enhancer, resulting in transcriptional upregulation [12]. Moreover, the IGF2/miRNA‐483 locus undergoes parental epigenetic imprinting, the loss of which unleashes transcription and correlates with increased CRC risk [13].

The molecular mechanisms by which miRNA‐483 exerts its protumorigenic functions in CRC are still obscure. So far, only miRNA‐483‐3p was partly characterized with the identification, by *in silico* prediction of 3'UTR RNA binding, of putative targets. These include the BH3‐containing protein BBC3/PUMA, whose downregulation prevents apoptosis in cell lines of various origins [14], and PARD3, whose targeting increases TGF‐β1‐induced invasion in anaplastic thyroid cancer [15].

To investigate the miRNA‐483‐3p biological effects and targets in CRC we took advantage of m‐colospheres endogenously expressing low or high miRNA‐483‐3p levels, which we subjected to forward and reverse genetic approaches. We found that miRNA‐483‐3p expression correlates with the induction of cell invasion, epithelial–mesenchymal transition [16], and increased stem properties. By transcriptomic analysis, we identified N‐myc downstream‐regulated gene (NDRG1) as a new and prominent miRNA‐483‐3p target. This is known as a metastasis suppressor capable of downregulating the EGFR family [17, 18]. We then found that metastatic CRC overexpressing miRNA‐483‐3p hyperactivates EGFR/ERBB3 signaling in a ligand‐independent manner, leading to constitutive activation of EMT via the AKT/GSK3β/EMT transcription factors axis.

These results contribute to explaining the increased aggressiveness and resistance to specific EGFR targeting of CRC overexpressing the *IGF2*/miRNA‐483 locus and provide a rationale for therapeutic strategies aimed at extensive EGFR family inhibition.

## 2 Materials and methods

### 2.1 Human patients

Patients were recruited in a prospective observational trial (ClinicalTrials.gov, https://clinicaltrials.gov/ct2/show/NCT03347318?term=001‐IRCC‐00IIS‐10&draw=2&rank=1, No. NCT03347318) approved by the Candiolo Cancer Institute Review Board on human experimentation (protocol No. 001‐IRCC‐00IIS‐10). The study was conducted according to the standards set by the Declaration of Helsinki. Written informed consent was obtained from all patients and all samples were deidentified. Patients' data were treated according to ethical requirements and GCP.

### 2.2 Animal models

Animal models, including xenopatients, i.e., mice transplanted with patient‐derived xenografts (PDX) of CRC liver metastasis surgical samples, and spheropatients, i.e., mice transplanted with m‐colospheres (see also Section 2.18), were generated according to ethical regulations, methodologies and protocols approved by the Italian Ministry of Health (permissions No. 223/2015‐PR and No. 806/2016‐PR). 5‐ to 6‐week‐old male NOD.CB17‐*Prkdc* ^*scid*^/NcrCr mice (NOD/SCID, RRID:IMSR_CRL:394, Charles River Laboratories, Calco, Italy), were used for all *in vivo* studies. Mice were housed at a maximum of 6 per cage with a 14‐h light/10‐h dark cycle with food and water *ad libitum*. Mice were monitored at a minimum of twice weekly for general performance status and euthanized when the volume of xenografts reached 1600 mm^3^, or they displayed signs of distress, or weight loss ≥ 20%.

### 2.3 M‐colosphere derivation

M‐colospheres were derived from PDX and cultured at clonal density in standard medium, including basal medium supplemented with human recombinant EGF [(20 ng·mL^−1^), Sigma‐Aldrich, St. Louis, MO, USA] and fibroblast growth factor 2 (FGF2) [(10 ng·mL^−1^), PeproTech, Thermo Fischer Scientific, Waltham, MA, USA], unless otherwise indicated, as previously described [5, 6, 7]. M‐colospheres were regularly checked for correspondence with the original patient tumor by using the PowerPlex16 Cell‐ID assay (Promega, Madison, WI, USA), based on the analysis of 16 genomic STR markers plus amelogenin.

### 2.4 Cell lines

Human CRC cell lines HCT116 (RRID:CVCL_0291), SW48 (RRID:CVCL_1724), and GP2D (RRID:CVCL_2450) were obtained from American Type Culture Collection (Manassas, VA, USA). HCT116 were cultured in RPMI‐1640 (Sigma‐Aldrich), and SW48 and GP2D in DMEM (Sigma‐Aldrich). Both media were supplemented with 10% fetal bovine serum (Thermo Fischer Scientific) and 2 mm l‐glutamine (Sigma‐Aldrich). Cells were kept at 37 °C in 5% CO_2_ and routinely tested for being mycoplasma free using a PCR‐based method. Cells were re‐authenticated every 6 months, by using the PowerPlex16 Cell‐ID assay (Promega) as above.

### 2.5 Quantitative real‐time PCR (qPCR)

Total RNA was extracted from m‐colospheres or 10 μm‐thick Formalin‐fixed Paraffin‐Embedded (FFPE) tissue sections using Maxwell® RSC miRNA Tissue Kit (Promega), according to the manufacturer's instructions. For mRNA analysis, cDNA synthesis was performed with High Capacity Reverse Transcriptase kit (Thermo Fisher Scientific), according to the manufacturer's instructions. Amplification was performed with ABI PRISM 7900 HT (Applied Biosystem, Thermo Fischer Scientific) using Taqman Probes (*IGF2IN*, *SNAI1*, *BMI*, *EZH2*, *NANOG*, *NDRG1*, *EGFR*, *ERBB2*, *ERBB3*, Thermo Fisher Scientific) or with specific SYBR Green chemistry using specific primer pairs (*CDH1*, *NDRG1*, and *PARD3*, Table S1). Gene expression was normalized vs. *UBC*, *GADPH*, *TBP*, *B2M*, and *ACTB* as endogenous controls. For miRNA‐483‐3p analysis, starting from total RNA, reverse transcription was performed with specific primers (Table S1) with a TaqMan microRNA reverse‐transcription kit (Thermo Fischer Scientific) according to manufacturer's instructions. The diluted RT‐PCR products were amplified using TaqMan® Universal Master Mix No Amperase UNG and TaqMan® miRNA assay (hsa‐miR‐483‐3p; Thermo Fisher Scientific) with ABI PRISM 7900 HT (Applied Biosystem). The miRNA‐483‐3p gene expression was normalized to RNU48 expression (Thermo Fischer Scientific). Relative expression was calculated by subtracting scaled CT values from the total 40 cycles or as fold change (2^−ΔΔct^). Numerical results were expressed as means ± SEM (*n* ≥ 3 independent experiments, Student *t*‐test).

### 2.6 Public dataset analysis

Gene expression and miRNA‐483‐3p expression data for correlation studies were obtained from the cBioPortal website http://linkedomics.org/data_download/TCGA‐COADREAD/ and from Cancer Cell Line Encyclopedia (https://portals.broadinstitute.org/ccle). Patients' mutational and survival data were obtained from https://cbioportal.org/datasets [Colorectal adenocarcinoma (TCGA Firehose Legacy)].

### 2.7 M‐colosphere transduction

M‐colospheres were dissociated to single‐cell suspensions and 1 × 10^5^ cells were transduced in 6‐wells plates with lentiviral vectors encoding pre‐miR483‐3p or antagomiRNA‐483‐3p (System Biosciences, Mountain View, CA, USA) or shRNAs‐hNDRG1 (Vector Builder GmbH, Neu‐Isenburg, Germany; Table S1) at a Multiplicity Of Infection (MOI) of 5. All constructs included a GFP reporter under the control of CMV and H1 promoters and infection efficiency was verified by fluorescent microscopy or facs analysis, and by qPCR, 72 h after infection.

### 2.8 Cell viability

M‐colospheres were dissociated and seeded in 96‐microtiter wells at the concentration of 700–1000 cells·100 μL^−1^ in basal medium supplied with EGF, FGF2, neuregulin 1 or without factors, as indicated. In some experiments, MM121 [also known as seribantumab, Creative Biolabs (Shirley, NY, USA), 50 ng·mL^−1^] [19] was added after seeding and every 48 h. ATP consumption was measured at days 0 and 4 with Cell Titer Glo® and GloMax 96 Microplate Luminometer (Promega) according to manufacturer's instructions. In each experiment the average relative luminescence values (*n* ≥ 6 technical replicates) were normalized vs. day 0 and fold changes were reported (*n* ≥ 3 independent experiments, mean ± SEM, ANOVA, Bonferroni Multicomparison test).

### 2.9 3D‐spheroid invasion assay

3D‐spheroid assays were performed by seeding m‐colospheres in a matrix composed of growth factor reduced matrigel (BD Biosciences, Franklin Lakes, NJ, USA) and collagen type I [20]. Invasion was monitored using Cytation 3 Cell Imaging Multi‐Mode Reader (BioTek Instruments, Agilent Technologies, Santa Clara, CA, USA). Morphometric analyses were performed with imagej software (RRID:SCR_003070, NIH, Bethesda, MD, USA). Invaded area was normalized vs. day 0 and fold changes were reported (*n* ≥ 3 independent experiments, mean ± SEM, ANOVA, Bonferroni multicomparison test and Kolmogorov–Smirnov test).

### 2.10 Western blot analysis

Total proteins were extracted using RIPA buffer supplemented with a protease inhibitor cocktail (Roche Life Science, Saint Louis, MO, USA), NaVO_3_ 1 mm and NaF 1 mm, sonicated, quantified by BCA (Pierce, Thermo Fischer Scientific, Waltham, MA, USA). Proteins (12–20 μg) were separated on SDS/PAGE 4–12% or 4–20% (Invitrogen, Thermo Fischer Scientific, Waltham, MA, USA) and blotted onto nitrocellulose membrane. After blocking, primary antibodies were incubated at the indicated concentrations (Table S2). After incubation with HRP‐conjugated secondary antibodies (Jackson Lab, Thermo Fischer Scientific, Bar Harbor, ME, USA; Table S2), enhanced chemiluminescence (Biorad, Segrate, Italy) was used for detection according to manufacturer's instructions and images were acquired with the ChemiDoc Touch™ Imaging System (Biorad) with image lab software. The results shown are representative of *n* ≥ 3 independent experiments.

### 2.11 Immunofluorescence and immunohistochemistry

Samples undergoing immunofluorescence were either Formalin‐Fixed, Paraffin‐Embedded tumor specimens or growing agnospheres. The latter were harvested, fixed 10 min with PFA 4% at 4 °C, washed in PBS, and suspended in bio‐agar for cyto‐inclusion (Bio‐Optica, Milano, Italy) at 42 °C, processed for inclusion in paraffin, and stained. Briefly, 3 μm sections were kept for 1 h at 65 °C, then deparaffinized, rehydrated, and subjected to antigen retrieval for 40 min at 95 °C in TRIS/EDTA buffer pH 9.5. After cool down, sections were permeabilized in TBS/Tween20 0.1% 10 min and blocked in BSA 5%/normal donkey serum 5% in TBS Tween 0.1% for 1 h as previously described [21]. Primary antibodies were incubated O/N at 4 °C at the indicated concentrations (Table S2). For immunofluorescence, fluorescent‐conjugated secondary antibodies (Alexa Fluor, Thermo Fischer Scientific) were incubated for 40 min at RT in TBS/Tween20 0.1%. Nuclei were counterstained with DAPI (Dako, Merck KgaA Darmstadt, Germany) and mounted with gel mount. Images were acquired using a LEICA SPEII confocal microscope, equipped with a 20× oil objective. Optical single sections were acquired with a scanning mode format of 1024 × 1024 pixels. Fluorochromes unmixing was performed by the acquisition of the automated‐sequential collection of multi‐channel images, to reduce spectral crosstalk between channels. For immunohistochemistry, an additional peroxidase blocking was performed in H2O2 3%/methanol 50% incubated for 20 min in the dark. Secondary antibodies were HRP‐conjugated (Dako, Agilent), and diaminobenzidine (DAB) substrate chromogen kit (Dako) was used for detection. Nuclei were counterstained with Hematoxylin and images were acquired through lasv4.2 software (Leica Microsystem, Wetzlar, Germany). Images are representative of at least three independent immunostainings.

### 2.12 *In vitro* limiting dilution assay (LDA)

M‐colospheres were dissociated and seeded at limiting dilution concentrations (1–100 cells·100 μL^−1^) in ultra‐low‐attachment 96‐well microtiters (Corning, Somerville, MA, USA). Wells with primary spheres with a diameter ≥ 100 μm were indicated as ‘positive tests’. elda software [22] (http://bioinf.wehi.edu.au/software/elda/) was used to calculate stem cell frequency. Means and 95% confidence intervals (CI) are shown (*n* ≥ 3 independent experiments).

### 2.13 Differentiation assay

M‐colospheres were seeded in Nunc® Lab‐Tek® Chamber Slide™ system (Lab‐Tek chamber slides, Permanox, C6932, Sigma‐Aldrich) at a density of 15 × 10^3^ cells per well, in basal medium supplemented with FBS 10% for 4 days [7].

### 2.14 RNA sequencing

Quant‐seq 3' mRNA sequencing was performed using total RNA from m‐colospheres. RNA was purified with RNeasy Micro Kit (Qiagen, Dusseldorf, Germany) and quantified with qubit 2.0 fluorimetric assay (Thermo Fisher Scientific). Libraries were prepared from 100 ng of total RNA with Quant‐Seq 3' mRNA‐Seq Library Prep Kit FWD for Illumina (Lexogen GmbH, Wien, Austria), followed by library quality control by screen tape High sensitivity DNA D1000 (Agilent Technologies). Libraries were sequenced on a NovaSeq 6000 sequencing system (Illumina Inc., San Diego, CA, USA) as previously described [23].

### 2.15 Analysis of Quant‐Seq RNA data

Analysis of RNA‐sequencing data was performed as previously described [23]. Briefly, Illumina novaSeq base call (BCL) files were transformed into fastq files through bcl2fastq (version v2.20.0.422, Illumina Inc.), and sequence reads were trimmed using bbduk software (bbmap suite 37.31, Joint Genome Institute, Walnut Creek, CA, USA). Alignment was performed on hg38 reference assembly (Ensembl Assembly 93) with star 2.6.0a (GPL v3, open source) and gene expression levels were determined with htseq‐count 0.9.1. Genes with an average number of cpm (counts per million) < 5 and Perc of duplicated reads > 20% were excluded from the ensuing analysis. Gene expression normalization and differentially expressed genes were identified by using edger [24]. Volcano plots were generated by using ggplot2 package in R statistical environment. RNA‐sequencing data were released in a public repository (GEO accession number: GSE209535) and are available in Table S3.

### 2.16 Dual‐Luciferase miRNA target assay

Dual‐Luciferase‐NDRG1‐3'UTR miRNA Target vectors (GeneCopoeia™, Rockville, MD, USA; Table S1) were designed to quantitatively evaluate miRNA direct activity on *NDRG1* 3′ UTR. *NDRG1* 3′ UTRs were inserted downstream of the firefly luciferase gene. Wild‐type and mutated *NDRG1* 3′ UTRs, with mutations in the binding sites, were, respectively, inserted into custom vectors HmiT120908‐MT06 (NM_001374847.1) and CS‐HmiT120908‐MT06‐01 (NM_001374847.1 with mutations: AAGAGTGA>TACTCACT, position 1417–1423; GGTCAGAGTGA>CCTCACTCACT, position 126–142). The corresponding vector containing only the Luciferase reporter gene CmiT000001‐MT06 (GeneCopoeia™) was used as transfection control. HCT116 and SW48 (miR‐483‐3p low), and GP2D (miR‐483‐3p high) cells were seeded at the concentration of 10^5^ cells·500 μL^−1^ and co‐transfected with Dual‐Luciferase‐NDRG1‐3'UTR vectors (WT or mutated, 0.2 μg per well), and with either miR‐483‐3p or AntagomiR‐483‐3p, respectively, and scramble controls (0.5 μg per well). The efficiency of transfection was monitored by GFP expression (for miRNA‐483‐3p, AntagomiR‐483‐3p, empty vector). After 24 h, cells were harvested and the dual‐luciferase reporter assay was performed according to the manufacturer's instructions (GeneCopoeia™). Luciferase assay signal was normalized vs. internal Luciferase Renilla control, measured using GeneCopoeia Dual‐Luc kit (LF001) and read on a Promega instrument.

### 2.17 *In situ* proximity ligation assay (PLA) on m‐colospheres

*In situ* proximity ligation assay (PLA) assays were performed using the Duolink™ *In Situ* Red Starter Kit Mouse/Rabbit (Sigma) according to the manufacturer's instructions. Briefly, m‐colospheres were harvested, fixed 10 min with PFA 4% at 4 °C, washed in PBS, suspended in bio‐agar for cyto‐inclusion (Bio‐Optica) at 42 °C, paraffin‐embedded and sectioned as previously described [19]. Sections were incubated with primary anti‐EGFR, anti‐ERBB2, and anti‐ERBB3 (Table S2) at 4 °C overnight followed by species‐specific secondary antibodies conjugated with oligonucleotides (PLA probes). Negative control was performed by the sole EGFR primary antibody. After ligation and amplification, the signal from each pair of PLA probes in close proximity (< 40 nm) was visualized as an individual red spot and analyzed by LEICA SPEII confocal microscope and lasv software. PLA Multicolor Reagent Pack PLA kits DUO96000 and *In Situ* Red Starter Kit Mouse/Rabbit DUO92101 were purchased from Sigma‐Aldrich, Duolink™.

### 2.18 *In vivo* m‐colospheres transplantation (spheropatients)

To assess tumorigenic potential, m‐colospheres were dissociated as single‐cell suspensions and counted with trypan blue to exclude dead cells. 10^5^ cells were resuspended in 50 μL of basal medium mixed 1 : 1 with growth factor reduced matrigel (BD Bioscience, Franklin Lakes, NJ, USA) and injected subcutaneously in the flank of 5–6 weeks old male NOD.CB17‐Prkdcscid/J mice (referred to as ‘spheropatients’). During the procedure anesthesia with 2.5% isofluorane in 100% oxygen at a flow rate of 1 L·min^−1^ was delivered to mice. Tumor growth was measured by caliper using the formula (*d*)^2^ × (*D*)/2, where *d* and *D* are the minor and the major tumor axis, respectively. Tumors grown at the injection site were explanted, formalin‐fixed, and paraffin‐embedded to undergo histopathological evaluation.

### 2.19 Statistical analysis

RNAseq analysis was performed with the fgsea package in r statistical environment version 3.6. All the other statistical analyses were performed using graphpad prism 8.0 software (RRID:SCR_002798; Dotmatics, San Diego, CA, USA). Statistical significance was determined with the following tests: qPCR: Welch's *t*‐test or one‐way ANOVA Bonferroni Multicomparison test, as indicated; cell viability: parametric Student *t*‐test or one‐way ANOVA; 3D‐spheroid assay: one‐way ANOVA Bonferroni Multicomparison test and Kolmogorov–Smirnov test; PLA: Kolmogorov–Smirnov test; tumor volume: two‐way ANOVA; patients' Kaplan–Meier survival analysis: Mantel–Cox (log‐rank) test. Pearson's correlation analysis was also used. Data were displayed as mean ± standard error of the mean (SEM) of at least two independent experiments. A *P* value < 0.05 or a false discovery rate (FDR) < 10% was considered significant.

## 3 Results

### 3.1 miRNA‐483‐3p is overexpressed in a subset of CRC tissues and m‐colospheres

**Fig. 1:**
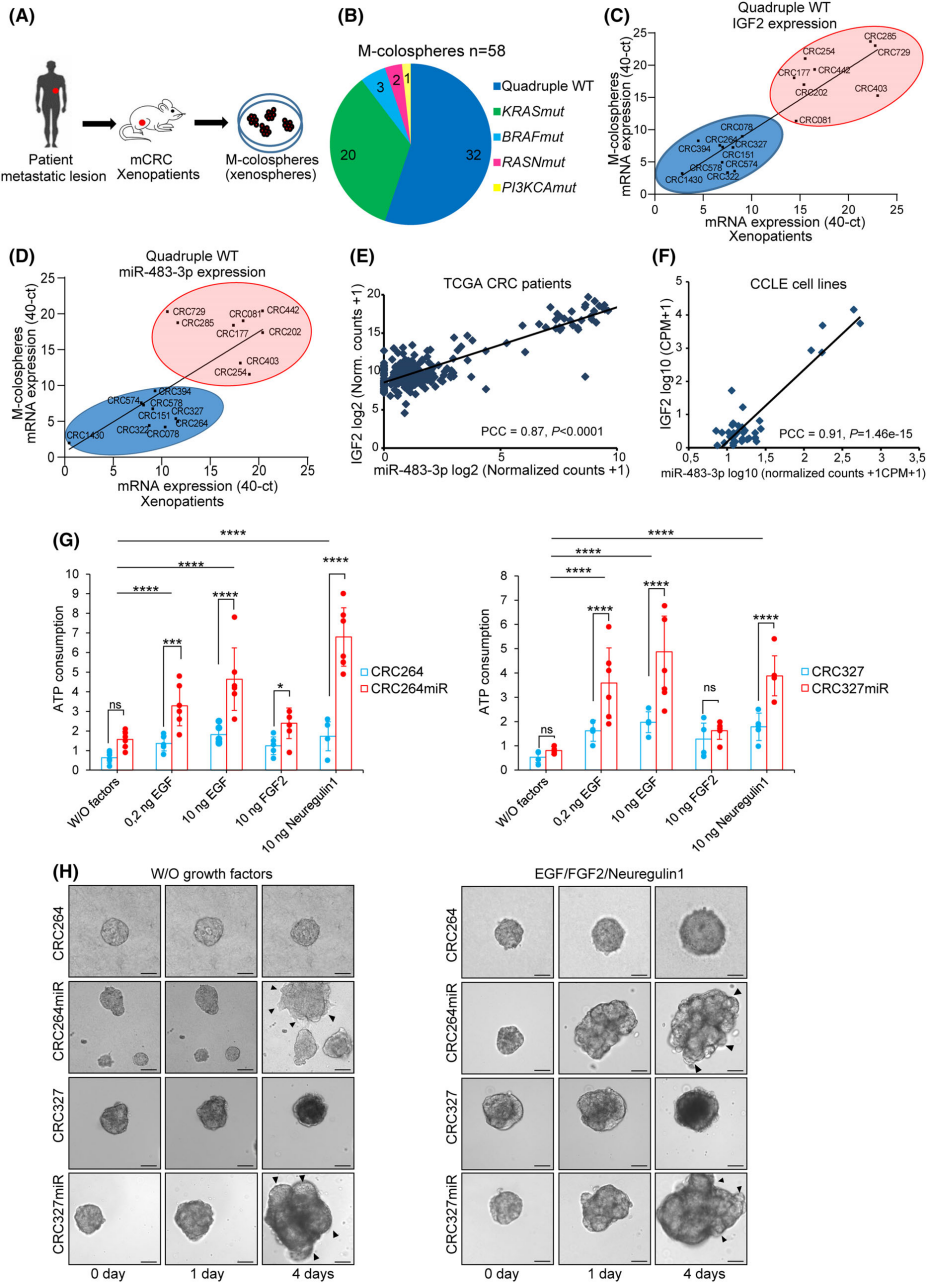
miRNA‐483‐3p is overexpressed in a subset of CRC patients' tissues and m‐colospheres and it sustains the invasive growth response to EGFR family ligands. (A) Generation of a molecularly annotated m‐colospheres biobank. M‐colospheres (also known as xenospheres) were derived from CRC metastasis samples previously implanted into immunocompromised mice and expanded as patient‐derived xenografts (xenopatients). (B) Pie charts showing the number of m‐colospheres displaying each indicated genetic alteration in the whole m‐colosphere panel (*n* = 58). WT, wild‐type; mut, mutation. (C, D) Pearson correlation of IGF2 expression (*r* = 0.8716, *P* < 0.0001, C) and miRNA‐483‐3p expression (*r* = 0.701, *P* = 0.0002, D), measured by real‐time qPCR, in quadruple WT m‐colospheres and matched xenopatients. (E, F) Pearson correlation coefficient (PCC) between the expression of miRNA‐483‐3p and IGF2 gene across TCGA colorectal cancer patients (*n* = 242, PCC = 0.87, *P* < 0.0001, E) and metastatic colorectal cancer cell lines of the Cancer Cell Line Encyclopedia (CCLE, *n* = 39, PCC = 0.91; *P* = 1.46e‐15, F), for which both RNA‐seq and miRNA‐483‐3p seq data were available (Table S4). (G) Cell viability of CRC264, CRC264miR, CRC327 and CRC327miR m‐colospheres kept for 4 days in basal medium (without growth factors: W/O factors), or in basal medium with EGF (0.2 or 10 ng·mL^−1^) or FGF2 (10 ng·mL^−1^) or neuregulin 1 (10 ng·mL^−1^). Bars: ATP consumption, fold change at day 4 vs. day 0 ± SEM (*n* > 7; ns, not significant; *, *P* < 0.005; ***, *P* < 0.001; ****, *P* < 0.0001; one‐way ANOVA). (H) 3D‐spheroid invasion assay. CRC264, CRC264miR, CRC327, CRC327miR m‐colospheres were embedded in a matrigel‐collagen type I matrix and their growth was monitored by time‐lapse microscopy at the indicated time points (*n* = 3). The dark core (4d) indicates necrosis. Arrowheads: cell protrusion or dissociation from the spheroid surface. Scale bar, 50 μm. Quantitative morphometric analysis is shown in Fig. S1F.

We previously generated a large cohort of human metastatic CRC (mCRC) xenografts (or xenopatients), from which we derived m‐colospheres (*n* = 58), also called ‘xenospheres’, i.e. cultures enriched in cells with cancer‐initiating properties (Fig. 1A,B) [5, 6]. Previous characterization showed that m‐colospheres retain the same driving genetic alterations as the matched xenopatients (Table S4) [6, 7]. To unveil novel genetic mechanisms that may support tumor aggressiveness, we focused on the ‘quadruple WT’ case subgroup, which is devoid of mutations in the RAS/phosphoinositide 3‐kinase (PI3K) pathway, and where correlations between oncogenic pathways and mechanisms of tumor progression are partly obscure [5, 6, 7]. Interestingly, in a previous study, we found that a subset of quadruple WT xenopatients displayed IGF2 overexpression and was only partially sensitive to EGFR‐targeted therapy with the EGFR antibody cetuximab [8]. In these tumors, IGF2 was shown to provide a direct contribution to bypass EGFR activity requirement [8]. Among available quadruple WT m‐colospheres (*n* = 17), 8/17 were derived from IGF2 overexpressing xenopatients and were confirmed to overexpress IGF2 (Fig. 1C and Table S4). In the remaining quadruple WT cases (*n* = 9/17), low levels of IGF2 expression reported in xenopatients were consistently observed in matched m‐colospheres (Fig. 1C and Table S4). Moreover, by extending IGF2 analysis to the entire cohort of available m‐colospheres (*n* = 33), including also those harboring RAS‐PI3K pathway mutations, we found that IGF2 expression levels remained consistent between m‐colospheres and matched xenopatients (Fig. S1A and Table S4).

Next, we showed that miRNA‐483‐3p expression levels were consistent with those of IGF2 in xenopatient tissues, indicating transcriptional co‐regulation of the two genes (Fig. S1B and Table S4). These results were corroborated by investigating the correlation between IGF2 and miRNA‐483‐3p expression across a panel of 242 colorectal cancer patients from The Cancer Genome Atlas [TCGA; Fig. 1E, Pearson Correlation Coefficient (PCC = 0.87, *P* < 0.0001)], and a panel of 39 colorectal cancer cell lines from the Cancer Cell Line Encyclopedia (CCLE, Fig. 1F and Table S5; PCC = 0.91; *P* = 1.46e‐15). In both datasets, a bimodal (low or high) and direct correlation between miRNA‐483‐3p and IGF2 mRNA expression was found. Importantly, in our cohort, miRNA‐483‐3p levels were faithfully retained from xenopatients to m‐colospheres (*r* = 0.6881, Fig. 1D and Fig. S1C) and identified two subgroups of m‐colospheres: (a) miRNA‐483‐3p‐low (represented by CRC078, CRC264, and CRC327), showing a typical epithelioid morphology, and (b) miRNA‐483‐3p‐high (represented by CRC254, CRC285, and CRC729), displaying a more mesenchymal phenotype, featuring loosely aggregated cells (Fig. S1D). These m‐colospheres were used to investigate the biological role of miRNA‐483‐3p with forward and reverse genetic approaches.

### 3.2 miRNA‐483‐3p overexpression sustains the invasive growth response to EGFR family ligands

We forced miRNA‐483‐3p expression in miRNA‐483‐3p‐low m‐colospheres CRC264 and CRC327 by lentiviral transduction, obtaining CRC264miR and CRC327miR. Both m‐colospheres displayed miRNA‐483‐3p levels comparable to those observed in miRNA‐483‐high m‐colospheres (Fig. S1E).

First, we investigated whether miRNA‐483‐3p overexpression affected the response to growth factors included in the standard medium conventionally used for colosphere selection and propagation (EGF and FGF2) and to the ERBB3 ligand neuregulin 1, which we previously identified as capable of sustaining long‐term propagation of quadruple WT colospheres [7]. After growth factor deprivation by culture in basal medium for 48 h, CRC264miR and CRC327miR displayed a remarkably increased proliferative response to EGF, supplied either at a minimally effective (0.2 ng·mL^−1^) or standard (10 ng·mL^−1^) concentration, and to neuregulin 1 (10 ng·mL^−1^) compared with their matched parental m‐colospheres, while they did not change their response to FGF2 (Fig. 1G).

To investigate the invasive response, we challenged m‐colospheres in 3D assays with a matrix composed of growth factor reduced matrigel and collagen type I [20]. Notably, CRC264miR and CRC327miR could grow and invade the surrounding matrix even in the complete absence of exogenous growth factors (i.e., in basal medium), while their matched parental m‐colospheres were unable to invade the matrix, either in the absence or in the presence of growth factors, and CRC327 even showed signs of internal necrosis (Fig. 1H and Fig. S1F,G). The addition of exogenous growth factors (EGF and FGF2 + neuregulin 1) significantly increased miRNA‐483‐3p overexpressing m‐colospheres invasiveness at the early time‐point (Fig. 1H and Fig. S1F), but it was almost ineffective on parental m‐colospheres (Fig. 1H and Fig. S1G).

Altogether, these data show that miRNA‐483‐3p overexpression fosters m‐colosphere invasive growth, by promoting the proliferative response to EGFR family ligands, and conferring the ability to invade the extracellular matrix either in the presence or in the absence of growth factors.

### 3.3 miRNA‐483‐3p promotes the EMT program in m‐colospheres

**Fig. 2:**
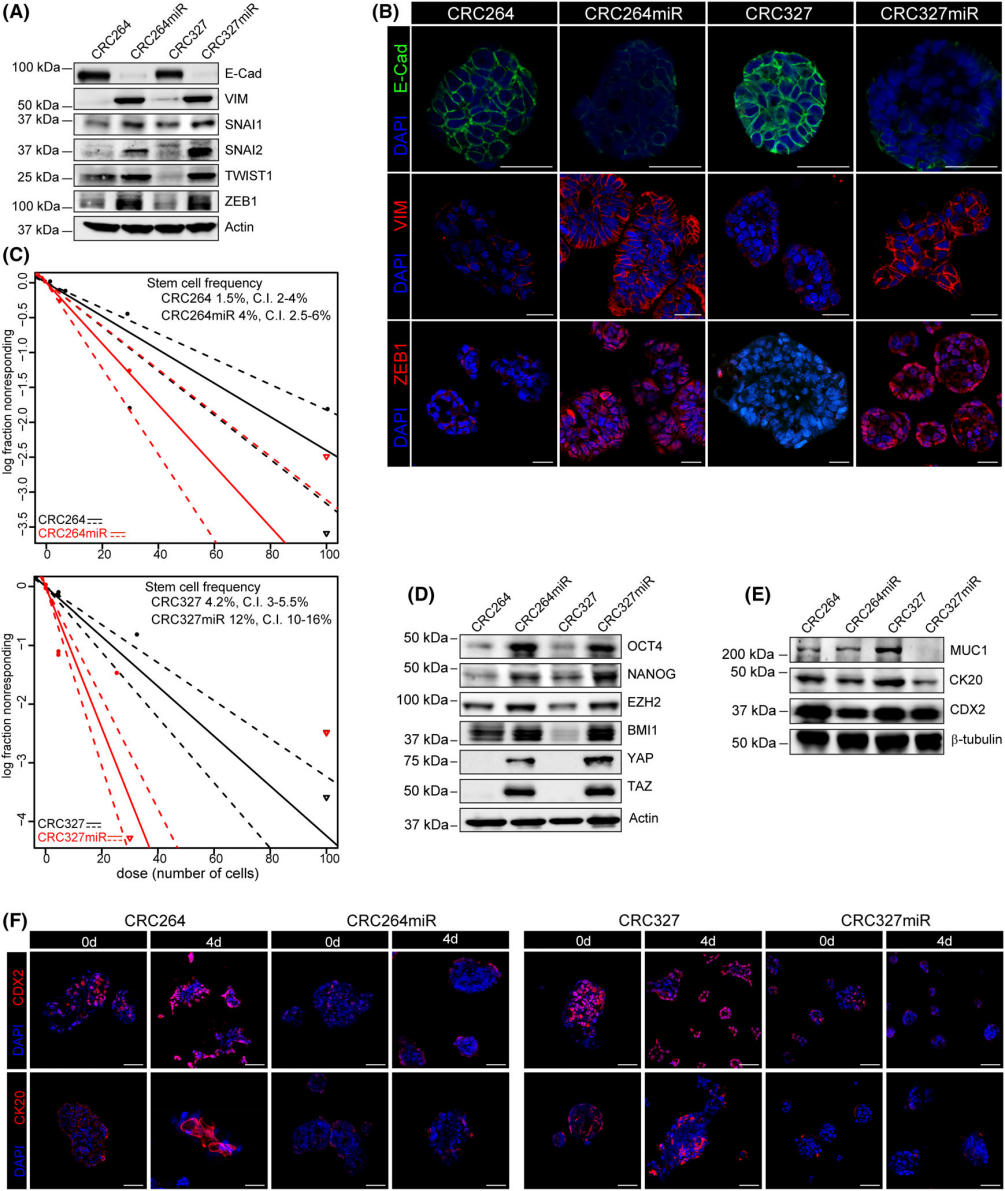
miRNA‐483‐3p promotes the EMT program and stem‐like traits in m‐colospheres. (A) Representative western blot analysis of EMT markers and core TFs in parental CRC264 and CRC327, and in miRNA‐483‐3p‐transduced m‐colospheres (CRC264miR and CRC327miR). Actin β was used as loading control. Densitometric analysis is shown in Fig. S2C (*n* ≥ 3). (B) Representative immunofluorescent stainings of E‐cadherin (E‐Cad), vimentin (VIM) and ZEB1. Nuclei were counterstained with DAPI. Scale bar, 50 μm (*n* ≥ 3). (C) *In vitro* limiting dilution sphere‐forming assay. For each m‐colosphere, plots generated by the elda software are shown, reporting the estimated stem cell frequency (percentage of clonogenic cells) with confidence intervals (C.I.). (D) Representative western blot analysis of stem cell TFs. Densitometric analysis is shown in Fig. S2D (*n* ≥ 3). (E) Representative western blot analysis of colorectal differentiation markers mucin 1 (MUC1), keratin 20 (CK20) and CDX2. Tubulin β was used as loading control (*n* = 3). Densitometric analysis is shown in Fig. S2D (*n* ≥ 3). (F) Differentiation assay. M‐colospheres were cultured for 4 days in pro‐differentiating conditions (adhesive substrate and basal medium containing 10% FBS). Immunofluorescent stainings for CDX2 and CK20 are shown. Nuclei were counterstained with DAPI. Scale bar: 50 μm (*N* = 3). Bright field acquisition is shown in Fig. S2E.

Next, we assessed whether the invasive growth phenotype displayed by m‐colospheres upon ectopic miRNA‐483‐3p expression co‐occurred with EMT program activation. Compared with their parental m‐colospheres, CRC264miR and CRC327miR, propagated in standard medium (i.e., in the presence of EGF and FGF2), displayed a looser epithelioid phenotype, similarly to native miRNA‐483‐3p‐high m‐colospheres (Fig. S2A,E). This trait correlated with E‐cadherin mRNA and protein downregulation and increased vimentin expression (Fig. 2A,B and Fig. S2B,C), a key EMT phenotypic hallmark [16]. Concurrently, protein levels of core EMT transcription factors (EMT‐TF), such as SNAI1, SNAI2, TWIST1, and ZEB1, were increased in CRC264miR and CRC327miR compared with their parental counterparts (Fig. 2A,B and Fig. S2C); notably, in miRNA‐483‐3p overexpressing m‐colospheres, TWIST1 and ZEB1 showed the highest increase, consistently with their prominent role in mCRC [25].

Altogether, these data unearth a correlation between miRNA‐483‐3p overexpression and EMT upregulation in mCRC.

### 3.4 miRNA‐483‐3p increases the stem‐like traits of m‐colospheres

A causal relationship between EMT program upregulation and maintenance of the stem phenotype has been shown in cancer cells [26, 27, 28, 29]. Accordingly, by *in vitro* clonogenic (limiting dilution) assays, miRNA‐483‐3p ectopic overexpression more than doubled the stem‐like cell frequency in m‐colospheres (CRC264miR = 4% vs. CRC264 = 1.5%, *P* = 6,72e‐04; CRC327miR = 8.5% vs. CRC327 = 4.2%, *P* = 3.77e‐05; Fig. 2C). Moreover, in miRNA‐483‐3p overexpressing m‐colospheres, we observed upregulation of stem core transcription factors such as OCT4, NANOG, EZH2, BMI1, YAP/TAZ (Fig. 2D and Fig. S2D) [30, 31, 32] and concomitant downregulation of colorectal differentiation markers such as mucin 1 (MUC1), keratin 20 (CK20) and CDX2 (Fig. 2E and Fig. S2D). CDX2 is both a differentiation marker and a transcriptional repression target of the SNAI family in the colonic epithelium [33, 34, 35].

Moreover, miRNA‐483‐3p ectopic overexpression prevented differentiation induced by switching m‐colosphere from stem culture (growth in suspension and standard medium) to seeding onto pro‐adhesive substrates and medium containing fetal bovine serum (10%) for 4 days [6, 36]. In such conditions, while parental CRC264 and CRC327 acquired differentiated traits including the formation of adherent flat colonies (Fig. S2E), and CDX2 and CK20 upregulation (Fig. 2F), CRC264miR and CRC327miR remained spheroid‐shaped (Fig. S2E) and displayed negligible CDX2 and CK20 levels (Fig. 2F).

These data indicate that miRNA‐483‐3p overexpression can sustain the stem‐like traits of m‐colospheres.

### 3.5 miRNA‐483‐3p directly targets the metastatic suppressor NDRG1

To uncover direct miRNA‐483‐3p targets responsible for sustaining the EMT/stem phenotype, we compared m‐colospheres overexpressing miRNA‐483‐3p with their respective parental counterparts by RNA sequencing (Table S3; GEO accession number: GSE209535). We first selected as significantly differentially expressed (DE) those genes with *P* < 0.001 (downregulated genes: *n* = 36 in CRC327miR vs. CRC327; *n* = 953 in CRC264miR vs. CRC264; upregulated genes: *n* = 100 in CRC327miR vs. CRC327; *n* = 216 in CRC264miR vs. CRC264). Next, to identify putative direct targets of miRNA‐483‐3p, we extracted genes that, starting from significant basal expression levels, were (a) significantly downregulated in both miRNA‐483‐3p‐overexpressing m‐colospheres (fold change < 0.55), and (b) contained computationally predicted miRNA‐483‐3p seed sequences at the 3'UTR (*n* = 6). *NDRG1* satisfied both requirements, being the most significantly downregulated in CRC327miR (Fc = 0.26, FDR = 1.99e‐09), and significantly downregulated in CRC264miR as well (Fc = 0.5, FDR = 3.79e‐09; Fig. S3A); importantly, by containing a miRNA‐483‐3p seed sequence at the 3'UTR, *NDRG1* was a likely direct miR‐483‐3p target (Fig. S3B, upper panel).

**Fig. 3:**
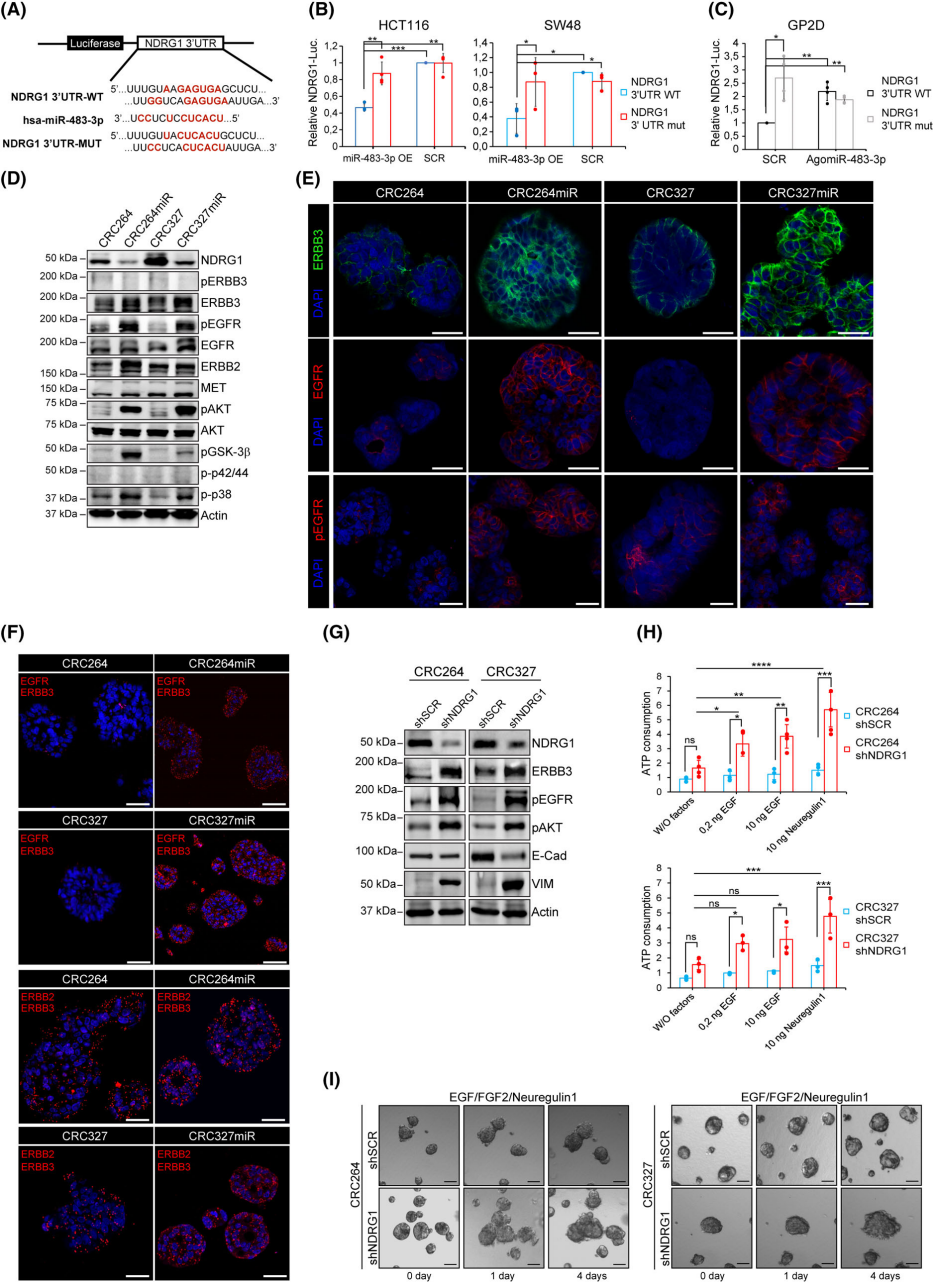
miRNA‐483‐3p targets the metastatic suppressor NDRG1, resulting in upregulation of EGFR family/AKT axis signaling. (A–C) Dual‐Luciferase miRNA target Assay. NDRG1‐3′UTR wt or mutated sequences were inserted downstream of the firefly luciferase gene, in Dual‐Luciferase miRNA target vectors; predicted miRNA‐483‐3p binding sites and relative mutations in 3'UTR are shown in red (A). HCT116 and SW48 (low endogenous miRNA‐483‐3p) were co‐transfected with Dual‐Luc vectors (*NDRG1*‐3'UTR WT: blue bars; or *NDRG1*‐3'UTR MUT: red bars) and with miRNA‐483‐3p (OE, overexpression) or miRNA‐483‐3p scrambled sequence (SCR) (B). GP2D (high endogenous miRNA‐483‐3p) were co‐transfected with Dual‐Luc vectors (*NDRG1*‐3'UTR WT: black bars; or *NDRG1*‐3'UTR MUT: gray bars) and AntagomiR‐483‐3p or scrambled sequence (SCR) (C). Bars represent Firefly vs. Renilla Luciferase (internal control) ratio. Signal ratio was normalized in each cell line vs. its *NDRG1*‐3'UTR + SCR control (error bars in (B and C) represent standard deviation; *, *P* < 0.05, **, *P* < 0.01; ***, *P* = 0.0001; *n* ≥ 3; Welch's *t*‐test). (D) Representative western blot of NDRG1, EGFR family receptors and downstream signal transducers in CRC264, CRC264miR, CRC327 and CRC327miR (*n* ≥ 3). pEGFR, phospho‐EGFR; pAKT, phospho‐AKT; pGSK‐3β(S9), phosphoSer9‐GSK3β; p‐p42/44, phospho‐p42/44; p‐p38, phospho‐p38. Densitometric analysis is shown in Fig. S3F (*n* ≥ 3). (E) Representative immunofluorescent stainings of ERBB3, and total and phosphorylated EGFR. Nuclei were counterstained with DAPI. Scale bar, 50 μm, (*n* ≥ 3). (F) Representative immunofluorescent stainings of Proximity ligation assay (PLA); red dots identify EGFR‐ERBB3 and ERBB2‐ERBB3 heterodimers (left). Scale bar, 50 μm. Quantification is shown in Fig. S3H (*n* = 3). (G) Representative western blot of NDRG1, EGFR family receptors, pAKT and EMT markers in CRC264shSCR, CRC264shNDRG1, CRC327shSCR and CRC327shNDRG1 (shSCR: control m‐colospheres transduced with scrambled shRNA; shNDRG1: transduced with shNDRG1). Densitometric analysis is shown in Fig. S3J (*n* ≥ 3). (H) Cell viability of CRC264shSCR, CRC264shNDRG1, CRC327shSCR and CRC327shNDRG1 m‐colospheres kept for 4 days in basal medium (without growth factors: W/O factors), or in basal medium with EGF (0.2 or 10 ng·mL^−1^) or neuregulin 1 (10 ng·mL^−1^). Bars: ATP consumption, fold change at day 4 vs. day 0 ± SEM (*n* > 3; ns, not significant; *, *P* < 0.05; **, *P* < 0.01; ***, *P* < 0.005; ****, *P* < 0.0001; one‐way ANOVA). (I) 3D‐spheroid invasion assay. CRC264shSCR, CRC264shNDRG1, CRC327shSCR and CRC327shNDRG1 m‐colospheres were embedded in a matrigel‐collagen type I matrix and their growth was monitored by time‐lapse microscopy at the indicated time points (*n* = 3). Scale bar, 50 μm. Quantitative morphometric analysis is shown in Fig. S3K.

A significant anticorrelation between *NDRG1* and miRNA‐483‐3p expression was confirmed in the panel of 39 CCLE colorectal cancer cell lines (*P* = 0.043, Fig. S3C and Table S5). To assess whether miRNA‐483‐3p directly targeted *NDRG1*, we performed luciferase reporter assays in two CRC cell lines expressing low endogenous miRNA‐483‐3p levels (HCT116 and SW48), and one expressing high endogenous miRNA‐483‐3p levels (GP2D; Table S5 and Fig. S3D). These cell lines expressed endogenous *NDRG1* mRNA levels that were anticorrelated with miR483‐3p, and they responded to miR‐483‐3p transfection with NDRG1 downregulation (HCT116 and SW48, Fig. S3D,E), or to AntagomiR‐483‐3p transfection with NDRG1 upregulation (GP2D, Fig. S3D,E). For reporter assays, we linked the luciferase coding sequence to *NDRG1*‐3'UTR, either wild‐type (WT) or mutated in the miRNA‐483‐3p predicted binding sites (Fig. 3A), and we transfected these constructs into HCT116 and SW48 cells, in the presence of miRNA‐483‐3p or scrambled sequence. We observed that the reporter activity of luciferase linked to *NDRG1*‐3'UTR WT, but not of luciferase linked to *NDRG1*‐3'UTR mutated, was significantly reduced by co‐transfection with miRNA‐483‐3p, and not by control scrambled miRNA (Fig. 3B). In the mirror experiment with GP2D cells, the reporter activity of luciferase linked to *NDRG1*‐3'UTR WT, but not of luciferase linked to *NDRG1*‐3'UTR mutated, was significantly increased by inhibition of endogenous miRNA‐483‐3p through co‐transfection of AntagomiR‐483‐3p, and not through control scrambled AntagomiR‐483‐3p (Fig. 3C).

Altogether, these data support the conclusion that NDRG1 is a direct target of miRNA‐483‐3p.

### 3.6 NDRG1 downregulation by miRNA‐483‐3p enhances the EGFR family/AKT axis signaling

The miRNA‐483‐3p target NDRG1 is particularly intriguing as it was identified as a metastasis suppressor in CRC [37] and in several other cancer types [38]. NDRG1 is a pleiotropic molecule with still unclear mechanistic functions; it has been shown to inhibit various oncogenic pathways, causing, among other events, decreased expression, activation, and heterodimerization of EGFR family receptors [39]. In particular, NDRG1 was shown to promote ERBB3 protein degradation by increasing its interaction with NEDD4 ubiquitin ligase [38, 40].

Therefore, we investigated whether NDRG1 downregulation in m‐colospheres transduced with miRNA‐483‐3p correlated with ERBB3 protein stabilization and activation of the downstream signaling pathways. Indeed, upon miRNA‐483‐3p ectopic overexpression, NDRG1 reduction correlated with increased ERBB3 protein levels (Fig. 3D and Fig. S3F) and stabilization at the plasma membrane, as observed by immunofluorescence (Fig. 3E), without effects on ERBB3 mRNA levels (Fig. S3G). Total EGFR protein or mRNA levels were not significantly increased (Fig. 3D and Fig. S3G), but EGFR was stabilized at the membrane, as well (Fig. 3E). CRC264miR and CRC327miR displayed intense EGFR tyrosine phosphorylation (Tyr1068), as compared with parental m‐colospheres (Fig. 3D,E and Fig. S3F), despite growth factors (EGF and FGF2) had been withdrawn for 24 h before the experiment. EGFR activation was accompanied by intense activating phosphorylation of AKT (Ser473) and its target GSK3β (Fig. 3D and Fig. S3F). The latter occurred at Ser9, responsible for GSK3β inhibition [41], which is known to prevent SNAI1 and SNAI2 targeting to protein degradation, thereby leading to EMT upregulation [42, 43]. Interestingly, phosphorylation of p38 mitogen‐activated protein kinase (MAPK), but not p42/44 MAPK, was increased as well (Fig. 3D).

Altogether, the signal transduction features of miRNA‐483‐3p overexpressing m‐colospheres are consistent with activation of the pathway downstream EGFR/ERBB3 heterodimers, leading to preferential PI3K/AKT axis stimulation. *In situ* Proximity Ligation Assay (PLA), detecting interactions at distances < 40 nm, confirmed a dramatic increase in EGFR/ERBB3 spontaneous heterodimerization (i.e., occurring in the absence of any exogenous growth factor), in both CRC264miR and CRC327miR vs. their matched parental m‐colospheres (Fig. 3F and Fig. S3H). ERBB2/ERBB3 heterodimerization was instead unaffected by miRNA‐483‐3p expression (Fig. 3F and Fig. S3H).

To assess whether NDRG1 targeting is essential for the biochemical and biological activities associated with miRNA‐483‐3p expression, we silenced NDRG1 expression in parental CRC264 and CRC327 by shRNA lentiviral transduction, obtaining CRC264shNDRG1 and CRC327shNDRG1 (Fig. 3G and Fig. S3J). We then investigated whether NDRG1 knockdown reproduced the same phenotype observed after miRNA‐483‐3p transduction. Indeed, compared with parental m‐colosphere controls, CRC264shNDRG1 and CRC327shNDRG1 showed (a) increased expression of ERBB3 and pronounced phosphorylation of EGFR and AKT (Fig. 3G and Fig. S3J); (b) marker expression switch from epithelial to mesenchymal (Fig. 3G and Fig. S3J); and (c) increased sensitivity to EGF family ligands. Such response was more pronounced to neuregulin 1 in both NDRG1‐silenced m‐colospheres, either in viability (Fig. 3H) or invasive growth assays (Fig. 3I and Fig. S3K). Therefore, in m‐colospheres, miRNA‐483‐3p overexpression is phenocopied by NDRG1 silencing, indicating that NDRG1 is an essential miRNA‐483‐3p target.

In summary, these data suggest that the link between miRNA‐483‐3p and EMT is embodied by a cascade of events originating from the inhibition of NDRG1 protein translation, resulting in ERBB3 protein stabilization and upregulation of EGFR/ERBB3 signaling, followed by increased AKT activity, GSK3β inhibition, SNAI1 and SNAI2 protein stabilization, and E‐cadherin transcriptional repression (Fig. S3L).

### 3.7 Selective ERBB3 inhibition dampens miRNA‐483‐3p‐induced invasive growth

After showing that miRNA‐483‐3p overexpressing m‐colospheres upregulate the EGFR/ERBB3 signaling pathway via NDRG1 downregulation, we investigated whether such pathway is essential for the invasive growth phenotype induced by miRNA‐483‐3p expression (or by selective NDRG1 silencing). In particular, we choose to specifically inhibit ERBB3 as (a) miRNA‐483‐3p m‐colospheres display markedly increased sensitivity to neuregulin 1, the ERBB3‐specific ligand (Fig. 1G) [44]; (b) miRNA‐483‐3p stabilizes ERBB3 expression at the cell membrane (Fig. 3E); (c) upon heterodimerization with EGFR, ERBB3 is known to be the main activator of the PI 3‐kinase pathway, by p85 recruitment to the cell membrane [44, 45], leading to AKT and GSK3β phosphorylation, which is prominent in miRNA‐483‐3p overexpressing m‐colospheres (Fig. 3D). We thus treated m‐colospheres with the ERBB3‐specific MM121 (seribantumab) inhibitory antibody, known to downregulate ERBB3 from the cell surface (Fig. S4A) [19, 46].

**Fig. 4:**
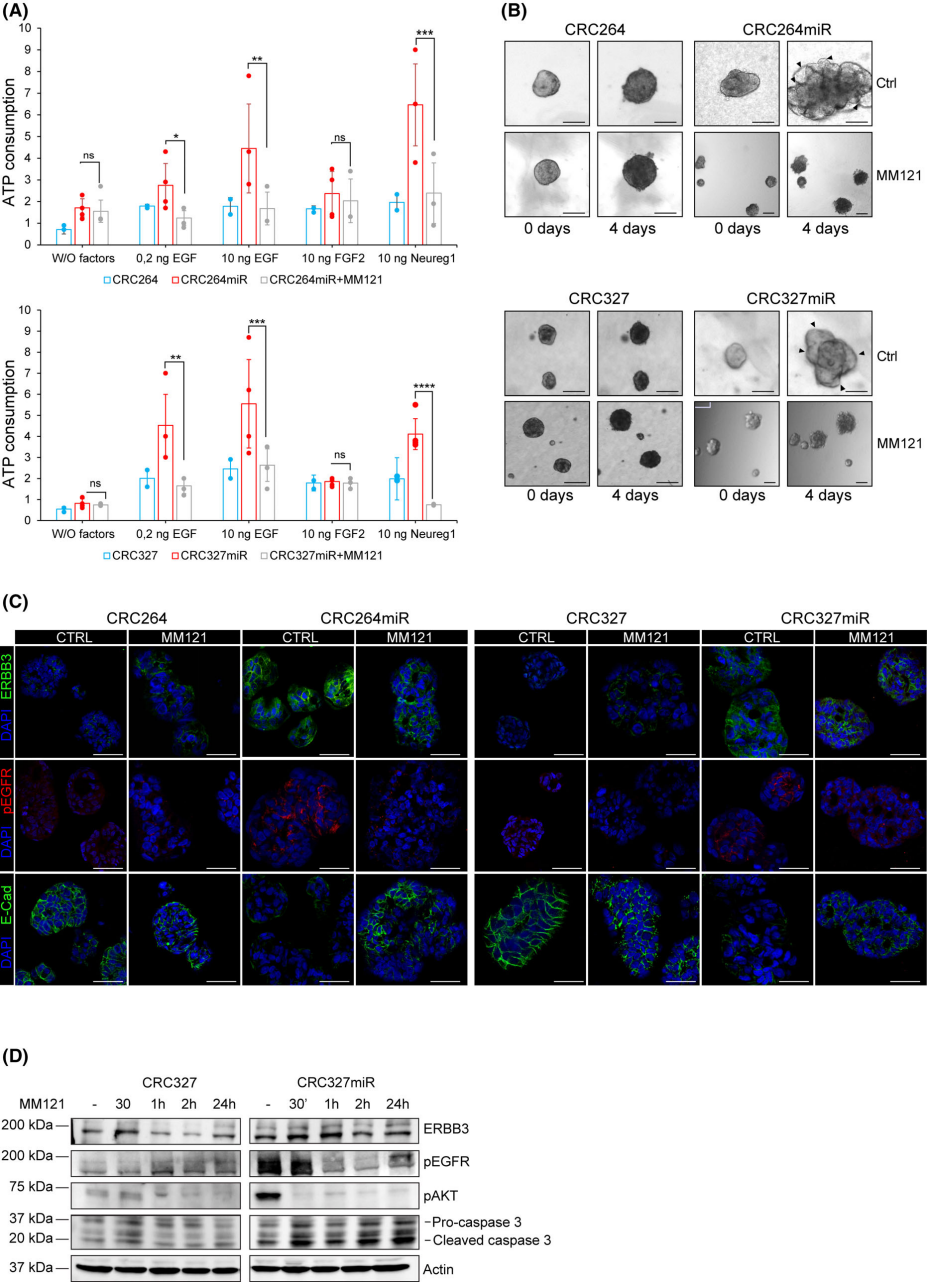
Selective ERBB3 inhibition dampens miRNA‐483‐3p‐induced invasive growth. (A) Viability of m‐colospheres (upper panel: CRC264 and CRC264miR; lower panel: CRC327 and CRC327 miR) kept for 4 days in basal medium (without growth factors: W/O factors), or in basal medium with EGF (0.2 or 10 ng·mL^−1^) or FGF2 (10 ng·mL^−1^) or neuregulin 1 (10 ng·mL^−1^). M‐colospheres overexpressing miRNA‐483‐3p were treated with MM121 (50 ng·mL^−1^). Bars represent ATP consumption, fold change at day 4 vs. day 0 ± SEM (*n* > 3; *, *P* = 0.05; **, *P* < 0.001; ***, *P* < 0.005; ****, *P* < 0.0001; one‐way ANOVA). (B) 3D‐spheroid invasion assay. M‐colospheres (upper panel: CRC264 and CRC264miR; lower panel: CRC327 and CRC327miR) were embedded in a matrigel‐collagen type I matrix in the absence of growth factors, with or without MM121 (50 ng·mL^−1^). Their growth was monitored by time‐lapse microscopy at the indicated time points (*n* = 3). Arrowheads: cell protrusion or dissociation from the spheroid surface. Scale bar, 50 μm. Quantitative morphometric analysis of invaded areas is shown in Fig. S4B. (C) Representative immunofluorescent stainings of ERBB3, phospho‐EGFR (pEGFR) and E‐cadherin in CRC264, CRC264miR, CRC327, CRC327miR m‐colospheres grown in absence or presence of MM121 (50 ng·mL^−1^) for 24 h. Nuclei were counterstained with DAPI. Scale bar, 50 μm (*n* = 3). (D) Representative western blot of ERBB3, pEGFR, pAKT, caspase 3 in CRC327 and CRC327miR after MM121 treatment (50 ng·mL^−1^). M‐colospheres were collected at the indicated time points (*n* = 3).

In cell viability assays, MM121 fully abolished the hyperproliferative effect of neuregulin 1, observed in CRC264miR and CRC327miR, reducing it to levels detectable in their matched parental m‐colospheres (Fig. 4A); moreover, MM121 significantly reduced also the response to EGF, which, like the response to neuregulin 1, was conceivably mediated by EGFR/ERBB3 heterodimers (Fig. 4A). In addition, MM121 fully prevented invasive growth promoted by miRNA‐483‐3p in m‐colospheres cultured in the absence of any growth factor, and induced the appearance of inner necrotic areas, as observed in 3D assays (Fig. 4B and Fig. S4B). Consistently, in miRNA‐483‐3p overexpressing m‐colospheres, MM121 downregulated ERBB3 from the cell surface, inhibited EGFR and AKT phosphorylation, and it induced E‐cadherin re‐exposure on the cell surface, as assessed by immunofluorescence (Fig. 4C) and western blot (Fig. 4D). Notably, together with inactivation of the EGFR/ERBB3/AKT pathway, MM121 caused robust induction of cleaved‐Caspase3 (Fig. 4D), indicating that not only proliferation and invasive growth associated with miRNA‐483‐3p were prevented (Fig. 4A,B), but apoptosis was induced as well.

These data support the key role of the ERBB3/EGFR axis in the phenotype of miRNA‐483‐3p overexpressing m‐colospheres, and indicate that ERBB3‐specific inhibition is sufficient to counteract this phenotype (Fig. S4A).

### 3.8 AntagomiRNA‐483‐3p upregulates NDRG1, impairs ERBB3 activity, and inhibits invasive and tumorigenic properties of m‐colospheres endogenously overexpressing miRNA‐483‐3p

**Fig. 5:**
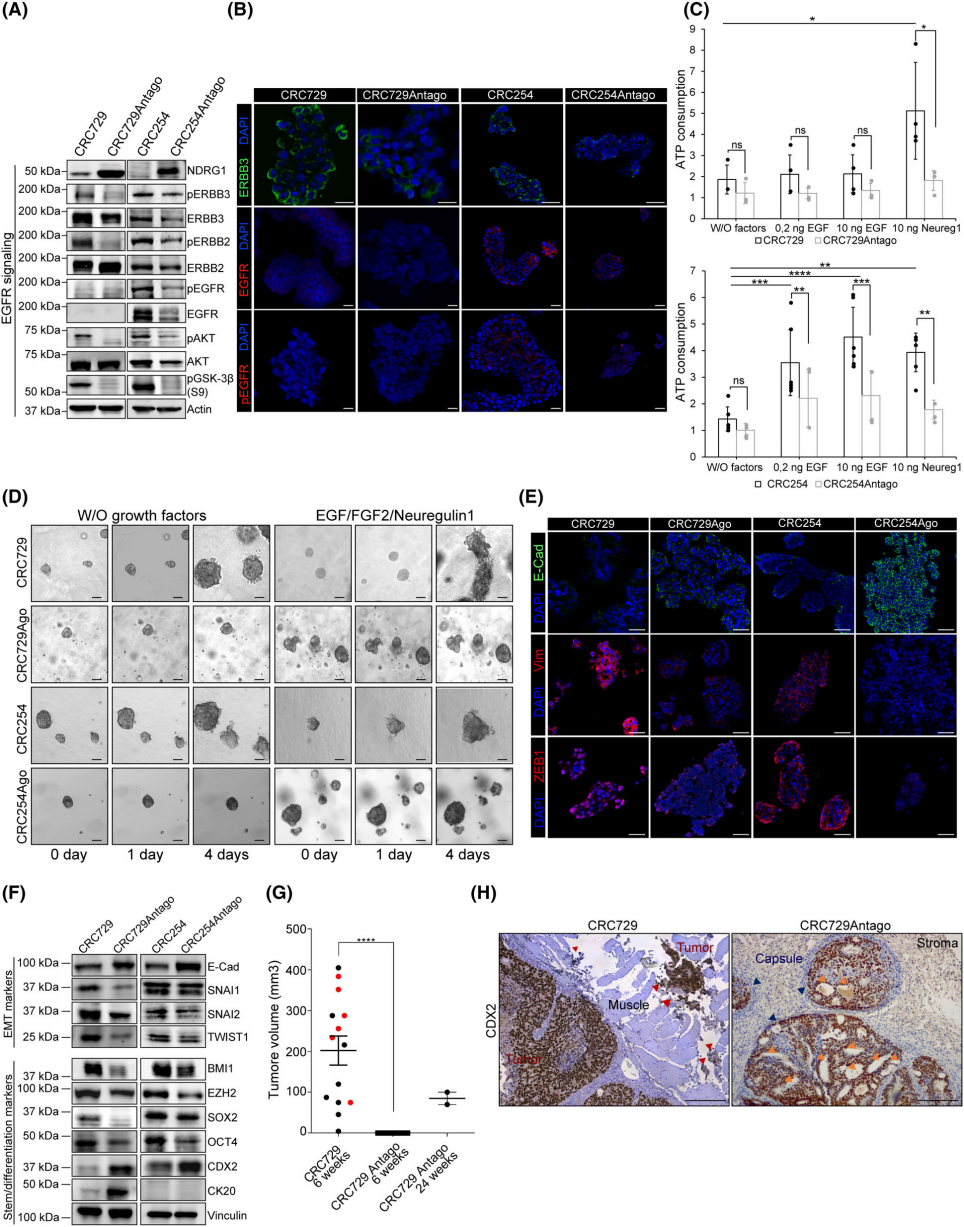
AntagomiRNA‐483‐3p reverts ERBB3 activity and inhibits tumorigenicity and invasion in m‐colospheres endogenously overexpressing miRNA‐483‐3p. (A) Representative western blot of NDRG1, EGFR family receptors and downstream signal transducers in CRC729, CRC254 and their counterparts transduced with antagomiRNA‐483‐3p (CRC729Antago and CRC254Antago). Densitometric analysis is shown in Fig. S5B (*n* = 3). (B) Representative immunofluorescent staining of ERBB3, EGFR, and phospho‐EGFR (pEGFR) in CRC729, CRC729Antago, CRC254 and CRC254Antago. Nuclei were counterstained with DAPI (*n* = 3). Scale bar, 50um. (C) Cell viability of CRC729, CRC729Antago, CRC254 and CRC254Antago m‐colospheres kept for 4 days in basal medium (without growth factors: W/O factors), or in basal medium with EGF (0.2 or 10 ng·mL^−1^) or neuregulin 1 (10 ng·mL^−1^). Bars: ATP consumption, fold change at day 4 vs. day 0 ± SEM (*n* > 7; ns, not significant; *, *P* < 0.05; **, *P* < 0.005; ***, *P* < 0.001; ****, *P* < 0.0001; one‐way ANOVA). (D) 3D‐spheroid invasion assay. CRC729, CRC729Antago, CRC254 and CRC254Antago m‐colospheres were embedded in a matrigel‐collagen type I matrix in the absence or in the presence of the indicated growth factors, and their growth was monitored by time‐lapse microscopy at the indicated time points Quantitative morphometric analysis is shown in Fig. S5C (*n* = 3). (E) Representative immunofluorescent staining of EMT markers E‐cadherin, vimentin and ZEB1 in CRC729, CRC729Antago, CRC254 and CRC254Antago. Nuclei were counterstained with DAPI (*n* = 3). Scale bar, 50 μm. (F) Representative western blot of EMT and colorectal stem/differentiation markers in CRC729, CRC729Antago, CRC254 and CRC254Antago. Densitometric analysis is shown in Fig. S5C (*n* = 3). (G) Tumor take and volume in mice subcutaneously transplanted with 10^5^ CRC729 (*n* = 14) or CRC729Antago (*n* = 14) m‐colosphere cells, measured at 6 or 24 weeks after injection (****, *P <* 0.0001; one‐way ANOVA). (H) Representative immunohistochemistry with human CDX2 antibody of tumors as in (G) (*n* = 3). CRC729: red arrowheads indicate cell clusters invading muscles; CRC729Antago: blue arrowheads indicate the presence of a stromal capsule surrounding tumor areas; orange arrowheads: adenomatous areas. Scale bar, 0.2 mm.

To further assess miRNA‐483‐3p and NDRG1 role in CRC EMT and invasive growth, by a complementary experimental approach we exploited CRC729 and CRC254 m‐colospheres, previously derived from CRC729 and CRC254 PDX [7], which express constitutively high miRNA‐483‐3p levels (Fig. 1D). These m‐colospheres show a highly aggressive and mesenchymal phenotype and *in vitro* independence from exogenous growth factors. However, CRC254 retains sensitivity to both exogenous EGF and neuregulin 1, while CRC729, lacking EGFR expression, responds only to neuregulin 1 [7]. In CRC729 and CRC254, we blocked miRNA‐483‐3p activity by transducing the miRNA‐483‐3p complementary sequence (antagomiRNA‐483‐3p), obtaining CRC729Antago and CRC254Antago. Effective miRNA‐483‐3p inhibition was verified by analysis of NDRG1 and an additional miRNA‐483‐3p target (PARD3) [15], which were consistently upregulated at mRNA level (Fig. S5A). NDRG1 protein, low in parental CRC729 and virtually absent in CRC254, was drastically increased after antagomiRNA‐483‐3p expression (Fig. 5A). Accordingly, NDRG1 upregulation was accompanied by decreased ERBB3 protein levels, full inhibition of ERBB3, and downregulation of EGFR and ERBB2 (Fig. 5A,B). EGFR family inhibition resulted in the loss of AKT and GSK3β (S9) phosphorylation, as observed by western blot (Fig. 5A and Fig. S5B) and immunofluorescence (Fig. 5B).

Consistently with the above protein expression and phosphorylation pattern, antagomiRNA‐483‐3p induced in CRC729 and CRC254 biological outcomes opposite to those induced by miRNA‐483‐3p expression in CRC264 or CRC327. In particular, antagomiRNA‐483‐3p decreased CRC729 and CRC254 proliferative rate, especially in the presence of EGF family ligands (Fig. 5C), and dampened their ability to invade the extracellular matrix (Fig. 5D and Fig. S5C). EMT inhibition by antagomiRNA‐483‐3p was further documented by induction of E‐cadherin expression and downregulation of EMT‐core TFs and vimentin, and of colorectal stem markers (Fig. 5E,F and Fig. S5B). Moreover, antagomiRNA‐483‐3p decreased CRC729 and CRC254 stem cell frequency, as observed in LDA assays (CRC729 = 6.6% vs. CRC729Antago = 3.8%, *P* = 0.0003; CRC254 = 12.5% vs. CRC254Antago = 3.3%, *P <* 0.0001; Fig. S5D), and abolished resistance to serum‐stimulated differentiation, as indicated by cell‐substrate adhesion and acquisition of flat morphology (Fig. S5E).

Finally, based on *in vitro* evidence, we investigated whether antagomiRNA‐483‐3p could counteract CRC729 growth and invasion *in vivo*. Therefore, we subcutaneously transplanted 5 × 10^5^ CRC729Antago or their parental counterpart in NOD/SCID mice (spheropatients). After 6 weeks, all 14 CRC729 spheropatients developed palpable grafts (average volume = 231 mm^3^), six of which (irrespectively of tumor volume) displayed underlying muscle invasion (Fig. 5G,H). Strikingly, none of the 14 CRC729Antago spheropatients developed a palpable tumor until 24 weeks after m‐colosphere injection (Fig. 5G). At this time‐point, two mice displayed tumors with a poorly aggressive aspect, featuring a more differentiated adenomatous morphology, the presence of a well‐defined connective capsule, and lack of peritumoral invasion (Fig. 5H).

### 3.9 miRNA‐483‐3p expression directly correlates with increased EMT and poor prognosis in CRC patients

**Fig. 6:**
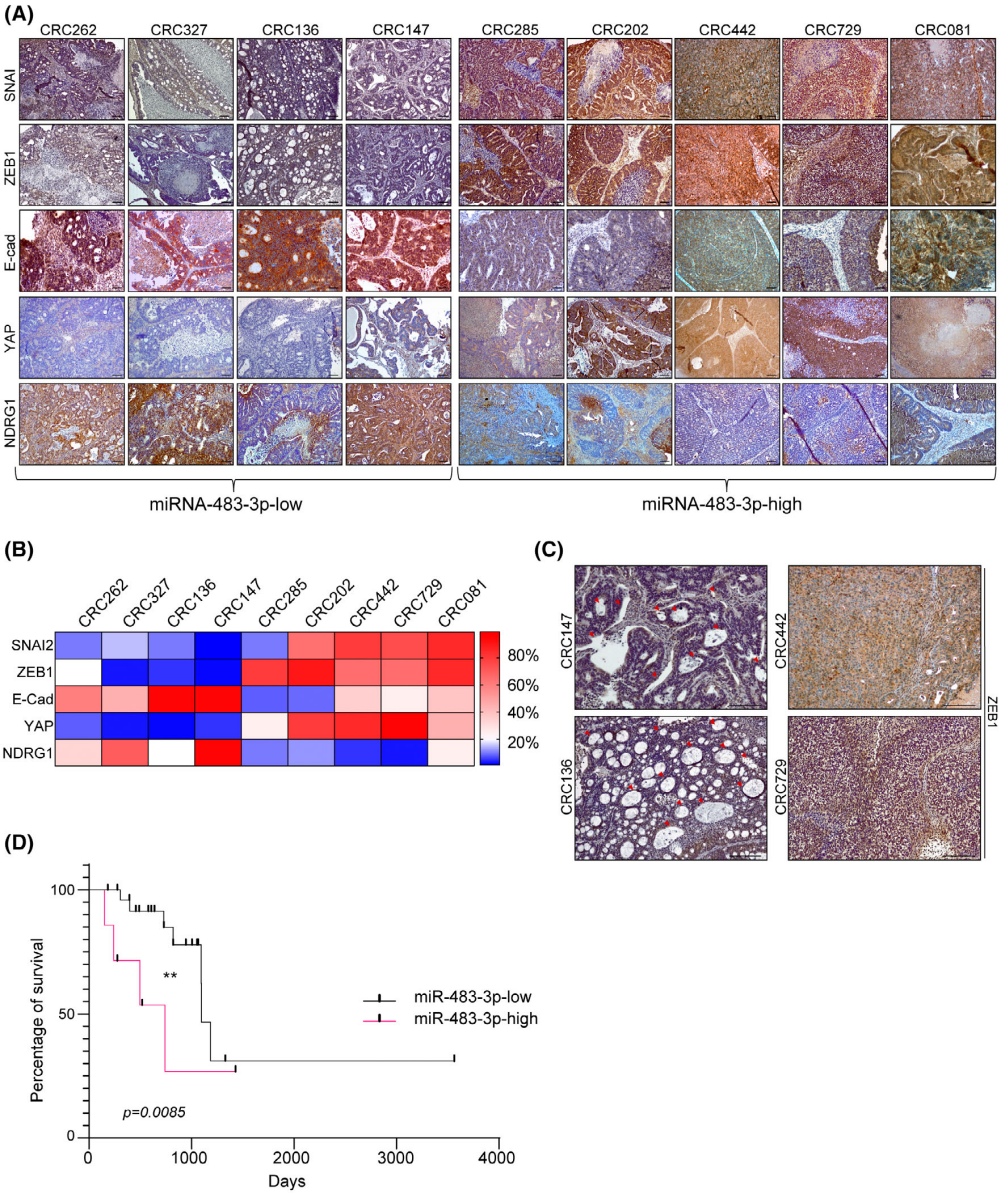
miRNA‐483‐3p expression directly correlates with increased EMT and poor prognosis in CRC patients. (A) Immunohistochemistry of xenopatient tumors expressing low or high miRNA‐483‐3p levels with NDRG1 and the indicated human EMT markers (*n* = 3, magnification: 4×). Scale bar, 50 μm. (B) Heatmap showing the percentage of positive area in tumor sections stained as in (A) and analyzed with imagej software (*n* = 3). Score was calculated performing deconvolution, by subtracting the hematoxylin and eosin background and setting the same threshold for all samples; values represent percentage of positive area from three different and noncontinuous fields of each slide (mean ± SEM, *n* ≥ 3 per each xenopatient; miRNA‐483‐3p‐high vs. miRNA‐483‐3p‐low, SNAI2: *P* = 0.0308; ZEB1: *P* = 0.0097; E‐Cad: *P* = 0.0079; YAP: *P* = 0.0097; NDRG1: *P* = 0.016). (C) Higher magnifications (10×) of xenopatient tumor sections stained for ZEB1. Red arrowheads indicate adenomatous areas. Scale bar, 0.2 mm (*n* = 3). (D) Survival curve of grade III and IV quadruple WT colorectal cancer patients (TCGA cohort) displaying normal/low miRNA‐483‐3p levels (miRNA‐483‐low, *n* = 24) or miRNA‐483‐3p overexpression (miRNA‐483‐high, *n* = 13; **, *P* = 0.0085; Mantel–Cox).

To further investigate the correlation between miRNA‐483‐3p expression and aggressive tumor behavior, in our metastatic CRC xenopatient cohort we compared a panel of cases displaying high miRNA‐483‐3p expression levels with a panel of cases expressing low levels, as defined by qPCR (Fig. S1B and Table S4). miRNA‐483‐3p‐high tumors showed higher expression of EMT‐TFs such as SNAI2, ZEB1, and YAP, and weaker E‐cadherin expression, as compared with miRNA‐483‐3p‐low cases (Fig. 6A,B). Strikingly, NDRG1 expression showed an inverse correlation with EMT markers, being low in miRNA‐483‐3p‐high xenopatients and high in miRNA‐483‐3p‐low xenopatients (Fig. 6A,B). Accordingly, high miRNA‐483‐3p expression correlated with loss of epithelial and adenomatous morphology, high cellularity, and poorly differentiated features (Fig. 6A,C).

Finally, we analyzed whether miRNA‐483‐3p expression levels correlated with different clinical outcomes of CRC patients. Indeed, in the TCGA cohort group including stage III and IV quadruple WT patients for which miRNA data were available, we observed that patients overexpressing miRNA‐483‐3p (*n* = 14) displayed a significantly reduced overall survival compared with those expressing normal/low levels of miRNA‐483‐3p (*n* = 23; *P =* 0.0085; Fig. 6D).

Overall, these results suggest that miRNA‐483‐3p expression is associated with aggressiveness and poor prognosis in metastatic CRC patients. Given the tight relationship between miRNA‐483‐3p expression and upregulation of the ERBB3/AKT/EMT pathway, these patients could be further investigated for eligibility to personalized therapies targeting the overall EGFR family or, specifically, ERBB3.

## 4 Discussion

In CRC, as well as in most tumors, tackling invasiveness, dissemination, and resistance to agents targeting deregulated proliferative mechanisms remains challenging [1]. In CRC, EGFR signaling plays a central role and, as a notable exception among targeted therapies, EGFR inhibition is beneficial even when EGFR is genetically intact, provided the downstream RAS pathway is not oncogenically activated [47, 48]. Conceivably, CRC strongly relies on physiological EGFR signaling, as this receptor is essential to sustain the high proliferative rate of intestinal stem cells, the putative CRC cells of origin, which pass on defined molecular traits to their neoplastic progeny [49, 50]. Moreover, the EGFR family includes three additional members (ERBB2, −3 and −4), often expressed by CRC cells and able to form any kind of functional heterodimers upon binding to two distinct ligand families (EGF‐like factors, binding to EGFR, and neuregulins binding to ERBB3 or −4) [44]. Therefore, selective inhibition of EGFR binding to EGF ligands, as attained by clinically approved EGFR antibodies (such as cetuximab), may easily encounter bypass mechanisms and primary resistance involving other members of the EGFR family [51].

On these premises, not surprisingly CRC may evolve multiple mechanisms to sustain constitutive EGFR family signaling. Here we provide evidence that one of such mechanisms can be miRNA‐483‐3p overexpression, which mainly results in upregulated expression and signaling activity of ERBB3, a prominent regulator of stem‐like cells in CRC [7] and in glioblastoma, where ERBB3 specifically sustains the PI3K/AKT axis [46]. As ERBB3 is a heterodimerization partner lacking intrinsic tyrosine kinase activity [44], its oncogenic activation should preferentially rely on mechanisms controlling its expression, rather than on structural alterations, and, to such purpose, aberrant oncogenic or oncosuppressor miRNAs are attractive candidates.

Interestingly, forced miRNA‐483‐3p expression not only enhanced the m‐colosphere basal and growth factor‐dependent proliferative rate but also EMT and the ability to invade 3D extracellular matrices, a recognized harbinger of metastatic ability [25].

Addressing the link between a microRNA and a biological outcome such as EMT and increased invasiveness may imply to consider a plethora of direct and indirect microRNA targets. However, we offer evidence that the thread leading from miRNA‐483‐3p to EMT chiefly involves NDRG1. First, by providing the first analysis of the global transcriptomic response to miRNA‐483‐3p expression and functional experiments, we show that NDRG1 is prominently, robustly, and directly downregulated among other genes. Most importantly, we provide evidence that NDRG1 silencing mimics the effects of miRNA‐483‐3p expression. This evidence strongly associates the miRNA‐483‐3p‐induced phenotype, featuring increased sensitivity to EGF ligands, EMT, and invasive growth, to the specific activity of the miRNA‐483‐3p target NDRG1.

NDRG1 has been strikingly associated with cancer metastasis and thus defined as a metastasis‐suppressor gene in several carcinomas, including CRC [38, 52]. However, the mechanisms by which NDRG1 sustains metastasis have been so far elusive and can be at least in part elucidated by findings presented in this study. NDRG1 was known to promote protein degradation, in particular of EGFR family members [38]. By the use of a specific ERBB3 antibody (MM121), which was able to fully revert EMT and invasion induced by miRNA‐483‐3p forced expression and the ensuing NDRG1 downregulation, we show that, in this context, ERBB3 is essential for EMT and invasion induction. This is not surprising, considering the preferential ability of this receptor to activate the PI3K/AKT pathway, leading to GSK3β inhibition and escape of EMT‐core transcription factors, such as SNAI1 and SNAI2, from the degradative pathway [41], as observed in m‐colospheres.

Interestingly, EMT induction by miRNA‐483‐3p correlates with the enhancement of *in vitro* stem features and resistance to differentiation. This is consistent with previous findings showing that EMT program execution, either during physiologic embryonic development, or pathological tumor invasion and metastasis, correlates with the acquisition of a stem‐like phenotype [26, 27, 28, 29]. Through its impact on this trait, miRNA‐483‐3p can cooperate with the Wnt/β‐catenin pathway, usually activated by APC loss in colorectal pathway and known to upregulate EMT [53], further fueling an *in vivo* aggressive phenotype.

The modality of miRNA‐483‐3p overexpression, which is genetically fixed through IGF2 locus amplification, or by other inheritable mechanisms, as indicated by overexpression preservation from the patient to the xenopatient, and from the latter to m‐colospheres, indicates that the mechanism is selectable under evolutive pressure. Overall, these findings provide additional evidence that the EGFR signaling pathway is essential for CRC progression and suggest that a possible cooperation between miRNA‐483‐3p and IGF2 in fostering EGFR family activity deserves further investigation. Notably, this study shows that ERBB3 signaling upregulation can sustain the escalation from local proliferation to invasive growth and metastasis. In this perspective, an extended EGFR family targeting strategy could be more beneficial than the inhibition of one single family member.

## 5 Conclusions

In this study, we reconstruct for the first time the chain of mechanistic events that link miRNA‐483‐3p to the upregulation of invasive growth in metastatic colorectal cancer. We show that miRNA‐483‐3p targets NDRG1, a known ‘metastasis suppressor’, thereby activating the ERBB3‐AKT signaling pathway. The latter sustains EMT and stem properties, hence promoting colorectal cancer invasive growth, a critical prerequisite for metastasis. Notably, the proinvasive activity of miRNA‐483‐3p can be countered by ERBB3 blockade through selective antibodies. These findings not only present a new mechanism that can fuel colorectal cancer progression but also provide a rationale for targeted therapeutic intervention.

## Conflict of interest

LT reports grants from Symphogen, Servier, Pfizer, Menarini, Merus, and Merck KGaA outside the submitted work. The other authors declare no conflict of interest.

## Author contributions

EC involved in conceptualization, experimental planning, methodology, formal analysis, investigation, visualization, and writing—original draft. GR involved in formal analysis, investigation, methodology, and visualization. FV involved in formal analysis, investigation, and methodology. GG involved in data curation, formal analysis, investigation, methodology, and visualization. ADA involved in investigation, formal analysis, and methodology. NC involved in investigation and methodology. FO involved in formal analysis and methodology. AI involved in formal analysis. RA involved in investigation and methodology. FS involved in resources and methodology. PL involved in conceptualization, investigation, and resources. PMC involved in resources, conceptualization, and funding acquisition. AB involved in resources, conceptualization, and funding acquisition. LT involved in resources, conceptualization, funding acquisition, and writing—review and editing. CB involved in conceptualization, investigation, supervision, resources, project administration, funding acquisition, and writing—review and editing.

### Peer review

The peer review history for this article is available at https://www.webofscience.com/api/gateway/wos/peer‐review/10.1002/1878‐0261.13408.

## Supporting information


**Fig. S1.** miRNA‐483‐3p is consistently expressed with IGF2 in CRC and sustains the response to EGFR family ligands.
**Fig. S2.** miRNA‐483‐3p promotes the EMT program and stem‐like traits in m‐colospheres.
**Fig. S3.** miRNA‐483‐3p targets the metastatic suppressor NDRG1, resulting in upregulation of EGFR family/AKT axis signaling.
**Fig. S4.** Selective ERBB3 inhibition dampens miRNA‐483‐3p‐induced invasive growth.
**Fig. S5.** AntagomiRNA‐483‐3p upregulates NDRG1, impairs ERBB3 activity, and inhibits invasive and tumorigenic properties in m‐colospheres endogenously overexpressing miRNA‐483‐3p.
**Table S1.** List of probes, primers, and lentiviral constructs.
**Table S2.** List of antibodies.
**Table S4.** Clinical and molecular data of metastatic colorectal cancer patients and m‐colospheres.
**Table S5.** Expression of miRNA‐483‐3p and IGF2 gene across 39 colorectal cancer cell lines from the Cancer Cell Line (CCL) Encyclopedia dataset.

**Table S3.** Dataset: differentially expressed genes in CRC264, CRC264miR, CRC327, and CRC327miR.

## Data Availability

The complete dataset related to RNAseq analysis (referring to Fig. S3A) is available as Table S3, and it is released in the Gene Expression Omnibus under the accession number GSE209535 (https://www.ncbi.nlm.nih.gov/geo/query/acc.cgi?acc=GSE209535).
