# Supplementary material for: MicroRNA 483‐3p overexpression unleashes invasive growth of metastatic colorectal cancer via NDRG1 downregulation and ensuing activation of the ERBB3/AKT axis
Source: Mol Oncol. 2023 Mar 19;17(7):1280–301. doi: 10.1002/1878-0261.13408 (PMC10323897; doi:10.1002/1878-0261.13408)
Supplement: Supplementary file 1 — Fig. S1. miRNA‐483‐3p is consistently expressed with IGF2 in CRC and sustains the response to EGFR family ligands. Fig. S2. miRNA‐483‐3p promotes the EMT program and stem‐like traits in m‐colospheres. Fig. S3. miRNA‐483‐3p targets the metastatic suppressor NDRG1, resulting in upregulation of EGFR family/AKT axis signaling. Fig. S4. Selective ERBB3 inhibition dampens miRNA‐483‐3p‐induced invasive growth. Fig. S5. AntagomiRNA‐483‐3p upregulates NDRG1, impairs ERBB3 activity, and inhibits invasive and tumorigenic properties in m‐colospheres endogenously overexpressing miRNA‐483‐3p. Table S1. List of probes, primers, and lentiviral constructs. Table S2. List of antibodies. Table S4. Clinical and molecular data of metastatic colorectal cancer patients and m‐colospheres. Table S5. Expression of miRNA‐483‐3p and IGF2 gene across 39 colorectal cancer cell lines from the Cancer Cell Line (CCL) Encyclopedia dataset. [file MOL2-17-1280-s001.pdf]

Supplementary Information

MicroRNA-483-3p overexpression unleashes invasive growth of metastatic colorectal cancer *via* NDRG1 downregulation and ensuing activation of the ERBB3/AKT axis

Candiello et al.

***Corresponding author**: E-mail: carla.boccaccio@ircc.it

Content:

1. Supplementary Figures
2. Supplementary Tables
3. Description of Table S3 (separate .xlsx file)

1. Supplementary Figures

Fig. S1.

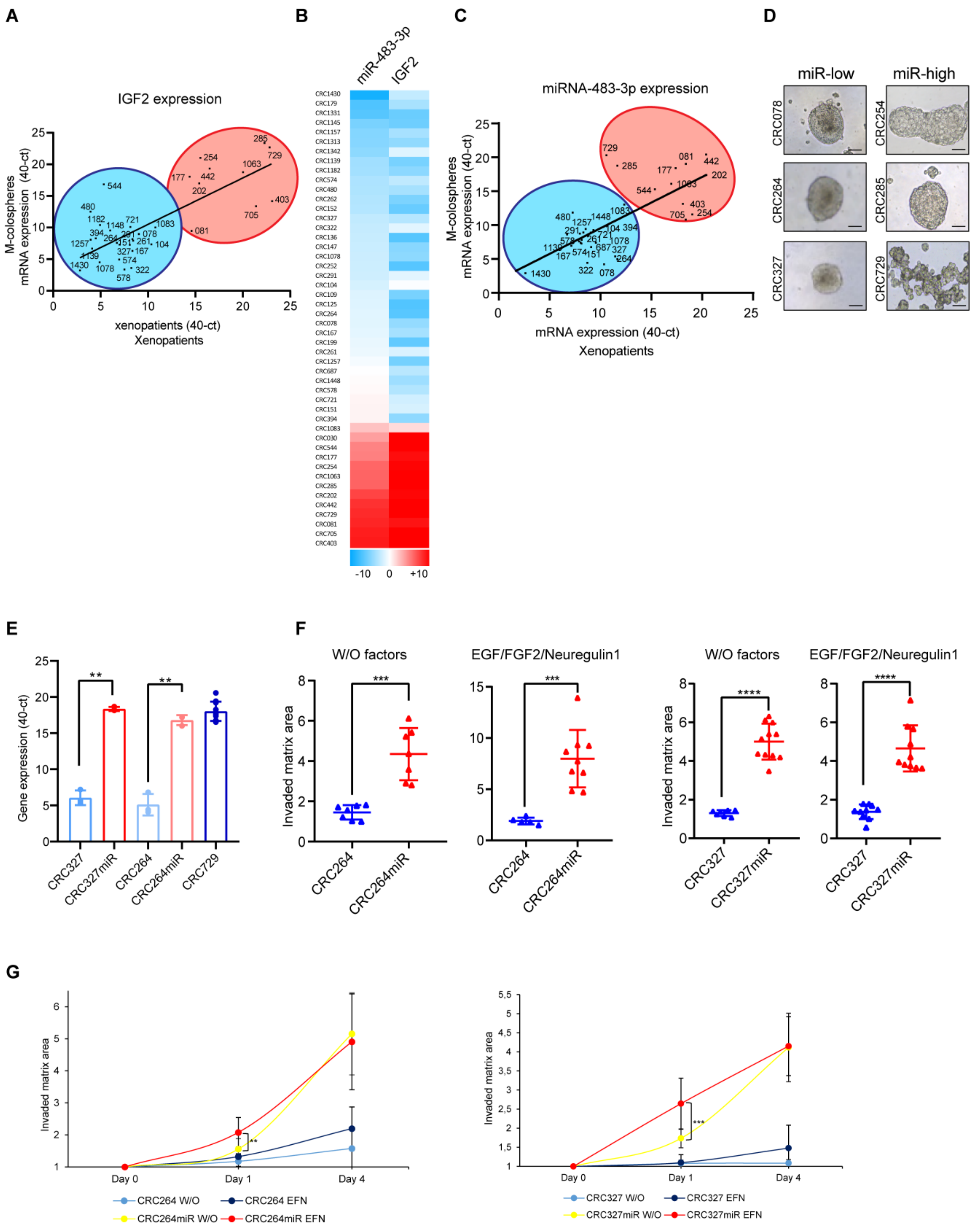

Fig. S1. miRNA-483-3p is consistently expressed with IGF2 in CRC and sustains the response to EGFR family ligands. **A** Pearson correlation of *IGF2* mRNA expression measured by qPCR in all available matched m-colospheres and xenopatients (n=33, Table S4), including quadruple WT (n=17) and RAS pathway mutated (n=16) (r=0.775 *P*<0.0001). **B** Heatmap showing consistent expression of miRNA-483-3p and *IGF2* in xenopatients (n=48, Table S4); for each sample, expression was calculated by subtracting scaled CT values from the total 40 cycles and represented as average-centered Log_2R. **C** Pearson correlation of miRNA-483-3p mRNA expression measured by qPCR in all available matched m-colospheres and xenopatients, including quadruple WT and RAS pathway mutated samples (r=0.688, *P*=0.0002). **D** Morphology of m-colospheres natively expressing low (left) or high (right) levels of miRNA-483-3p. Scale bar: 50 μm. **E** Bar graph showing miRNA-483-3p gene expression in m-colospheres transduced with a lentiviral vector encoding miRNA-483-3p vs their parental counterpart. CRC729, endogenously expressing high miRNA-483-3p levels, was used as internal control (CRC327miR vs CRC327: **, *P*=0.0012; C264miR vs CRC264: **, *P*=0.0015; Welch's *t* test). **F, G** Morphometric analyses of areas invaded by spheroids in 3D assays shown in Fig. 1F (n = 3). Box plots **F** represent fold change of areas invaded by each spheroid (dots) at day 4 vs day 0 (n = 3) (left: CRC264miR vs CRC264, w/o factors: ***, *P*=0.0006; CRC264miR vs CRC264, EFN: ***, *P*=0.0002; right: CRC327miR vs CRC327, w/o factors: ****, *P*<0.0001; CRC327miR vs. CRC327, EFN: ****, *P*<0.0001; ANOVA Bonferroni Multicomparison test). Curves **G** represent fold change (±SEM) of areas invaded by spheroids at different time points in the presence (EFN) or in the absence (w/o) of growth factors (Day 1, CRC264miR EFN vs CRC264miR W/O: **, *P*=0.0028; CRC327miR EFN vs CRC327miR W/O: ***, *P*=0.0004; ANOVA Bonferroni Multicomparison test). EFN: EGF, FGF2 and neuregulin1.

Fig. S2.

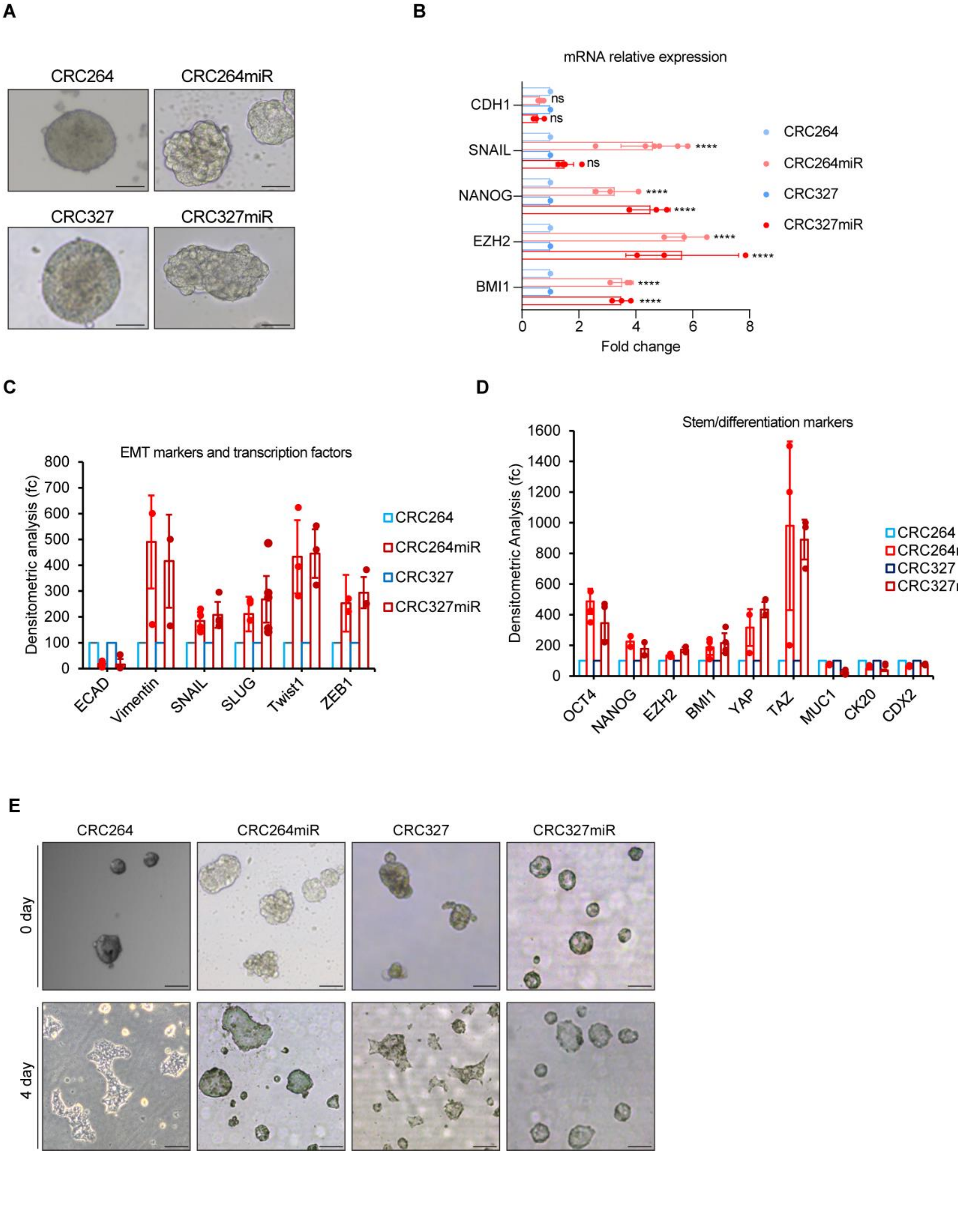
A
CRC264
CRC264miR
CRC327
CRC327miR
B
mRNA relative expression
CDH1
SNAIL
NANOG
EZH2
BMI1
ns
****
Fold change
CRC264
CRC264miR
CRC327
CRC327miR
C
EMT markers and transcription factors
Densitometric analysis (fc)
ECAD
Vimentin
SNAIL
SLUG
Twist1
ZEB1
D
Stem/differentiation markers
Densitometric Analysis (fc)
OCT4
NANOG
EZH2
BMI1
YAP
TAZ
MUC1
CK20
CDX2
E
0 day
4 day

Fig. S2. miRNA-483-3p promotes the EMT program and stem-like traits in m-colospheres. **A** Morphology of CRC264miR, CRC327miR and their respective parental m-colospheres. CRC264miR image is a higher magnification part of CRC264miR image shown in panel 2E. Scale bar: 50 µm. **B** Bar graph showing mRNA relative expression (measured by qPCR) of the indicated EMT and stem markers in CRC264miR and CRC327miR vs their respective parental m-colospheres (****, $P<0.0001$, one-way ANOVA Bonferroni Multicomparison test). *CDH1*: E-cadherin. **C, D** Densitometric analyses of western blots showing EMT core transcription factors (**C**, related to Fig. 2A), and stem and colorectal differentiation markers (**D**, related to Fig. 2D and E) in CRC264, CRC264miR, CRC327 and CRC327miR (n > 3, values were normalized vs internal loading controls and vs respective parental m-colospheres). **E** Bright field microscopy acquisition of differentiation assays; m-colospheres were cultured for 4 days in pro-differentiating conditions (adhesive substrate and basal medium containing 10% FBS). Scale bar: 100 µm.

Fig. S3.

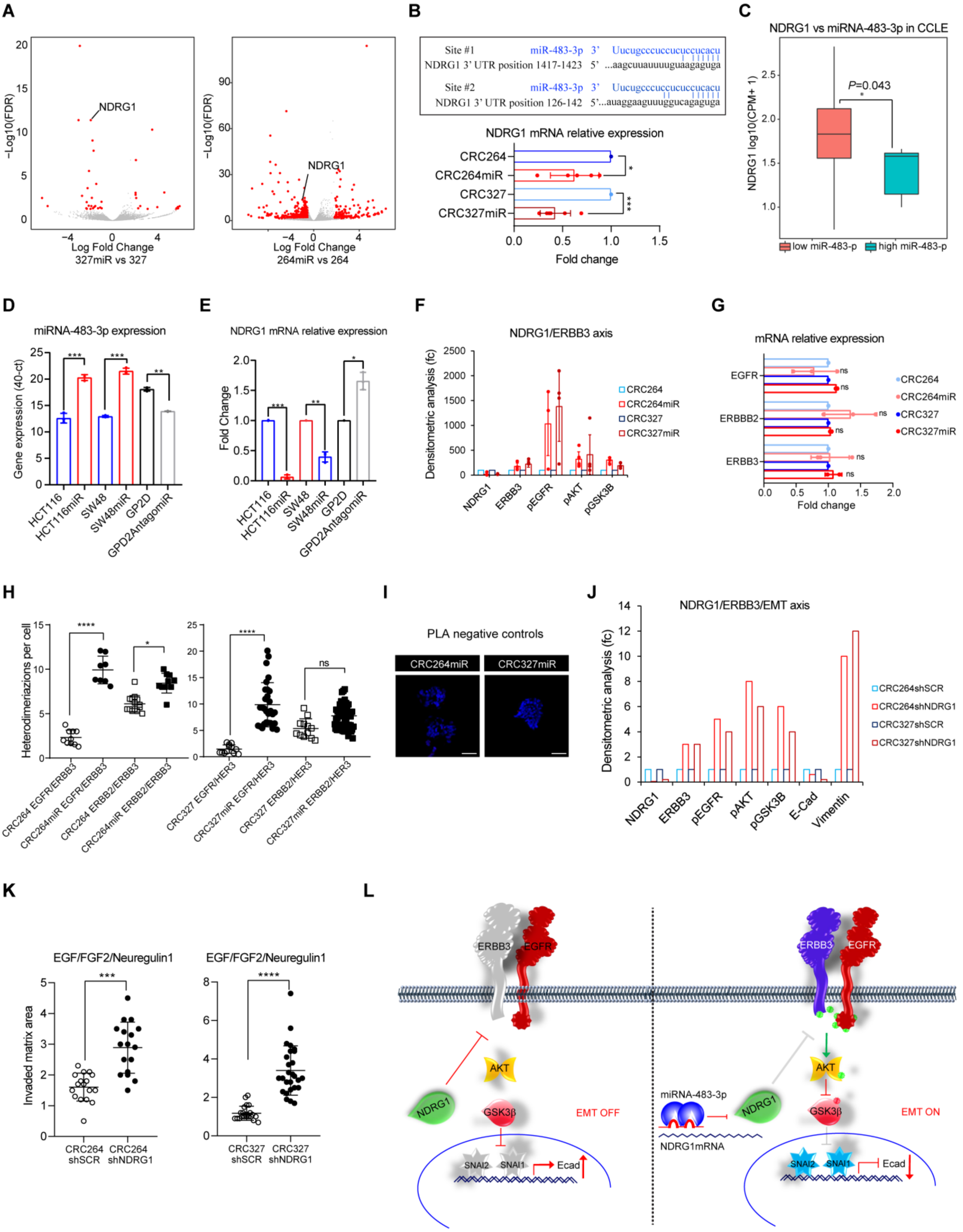

A
NDRG1
Log Fold Change
327miR vs 327
264miR vs 264
−Log10(FDR)
B
Site #1 miR-483-3p 3' Uucugcccucccucccucacu
NDRG1 3' UTR position 1417-1423 5' ...aagcuuauuuuguaagaguga
Site #2 miR-483-3p 3' Uucugcccucccucccucacu
NDRG1 3' UTR position 126-142 5'...auaggaaguuuggucagaguga
NDRG1 mRNA relative expression
CRC264
CRC264miR
CRC327
CRC327miR
Fold change
C
NDRG1 vs miRNA-483-3p in CCLE
P=0.043
NDRG1 log10(CPM+ 1)
low miR-483-p
high miR-483-p
D
miRNA-483-3p expression
Gene expression (40-ct)
HCT116
HCT116miR
SW48
SW48miR
GP2D
GPD2AntagomiR
E
NDRG1 mRNA relative expression
Fold Change
F
NDRG1/ERBB3 axis
Densitometric analysis (fc)
NDRG1
ERBB3
pEGFR
pAKT
pGSK3B
G
mRNA relative expression
EGFR
ERBBB2
ERBB3
Fold change
H
Heterodimerizions per cell
CRC264 EGFR/ERBB3
CRC264miR EGFR/ERBB3
CRC264 ERBB2/ERBB3
CRC264miR ERBB2/ERBB3
CRC327 EGFR/HER3
CRC327miR EGFR/HER3
CRC327 ERBB2/HER3
CRC327miR ERBB2/HER3
I
PLA negative controls
J
NDRG1/ERBB3/EMT axis
E-Cad
Vimentin
CRC264shSCR
CRC264shNDRG1
CRC327shSCR
CRC327shNDRG1
K
EGF/FGF2/Neuregulin1
Invaded matrix area
CRC264 shSCR
CRC264 shNDRG1
CRC327 shSCR
CRC327 shNDRG1
L
ERBB3
EGFR
AKT
GSK3β
SNAI2
SNAI1
Ecad
EMT OFF
EMT ON
miRNA-483-3p
NDRG1mRNA

Fig. S3. miRNA-483-3p targets the metastatic suppressor NDRG1, resulting in upregulation of EGFR family/AKT axis signaling. **A** Differentially regulated genes in CRC327miR vs CRC327 and CRC264miR vs CRC264; x-axis: fold change (log2); y-axis: False Discovery Rate (FDR, -log10). Red dots indicate genes with fold change<0.55 or >1.8 and FDR<0.005. **B** miRNA-483-3p seed sequences in the *NDRG1* 3'UTR (upper panel). Relative *NDRG1* mRNA expression in CRC264miR and CRC327miR vs the corresponding parental m-colospheres (n≥3, *, *P*=0.0281; ***, *P*=0.0003; Welch's *t* test; lower panel). **C** *NDRG1* mRNA expression in 39 colorectal cancer cell-lines from Cancer Cell Line Encyclopedia (CCLE) with available RNA-seq and miRNA-seq data, grouped into high vs low miRNA-483-3p expression (*P*=0.043, two tail Wilcoxon test). **D, E** Bar graphs showing miRNA-483-3p (D) and *NDRG1* (E) mRNA expression in CRC cell lines. HCT116 and SW48 cells, expressing low endogenous miRNA-483-3p levels, were transfected with miRNA-483-3p; GP2D cells, expressing high endogenous levels of miRNA483-3p, were transfected with AntagomiR-483-3p sequence. D, miRNA-483-3p expression: HCT116 vs HCT116miR: ***, *P*=0.0007; SW48 vs SW48miR: ***, *P*=0.0005; GP2D vs GP2DAntagomiR: **, *P*=0.0019. E, *NDRG1* relative expression, fold change: HCT116 vs HCT116miR: ***, *P*=0.0005; SW48 vs SW48miR: **, *P*=0.0063; GP2D vs GP2DAntagomiR: *, *P*=0.0157; Welch's test, n=3. **F** Densitometric analyses of western blots shown in Fig. 3D (n = 3). **G** mRNA relative expression of EGFR family receptors in CRC264miR and CRC327miR vs the respective parental m-colospheres (ns: not significant, one-way ANOVA). **H** Quantification of Proximity Ligation Assays (PLA) shown in Fig. 3F. Box plots indicate the number of red dots (EGFR/ERBB3 or ERBB2/ERBB3 heterodimers) per cell in CRC264 and CRC264miR (left) and CRC327 and CRC327miR (right) (ns, not significant; *, *P*=0.0127; ****, *P*<0.0001; Kolmogorov-Smirnov test). **I** PLA negative controls stained by one single primary antibody (anti-EGFR). Nuclei were counterstained with DAPI. Scale bar, 50 μm. **J** Densitometric analysis of western blots shown in Fig. 3G (n = 3). **K** Quantification of invasion assays shown in Fig. 3I. Box plots represent fold change of areas invaded by each spheroid (dots) at day 4 vs day 0 (n = 3). Left: C264shSCR vs CRC264shNDRG1: ***, *P*=0.0004. Right: CRC327shSCR vs CRC327shNDRG1: ** *P*=0.0016; ANOVA Bonferroni Multicomparison test. **L** Signaling events in the absence (left) or in the presence (right) of miRNA-483-3p. In the absence, NDRG-1 inhibits EGFR family signaling, preventing E-cadherin transcriptional repression and EMT (EMT OFF). In the presence of miRNA-483-3p, EGFR family signaling is unleashed, leading to AKT activation, GSK3-β inhibition, SNAI1 and SNAI2 stabilization, E-cadherin repression and EMT upregulation (EMT ON). Green stars: activating phosphorylation; red stars: inhibitory phosphorylation.

Fig. S4.

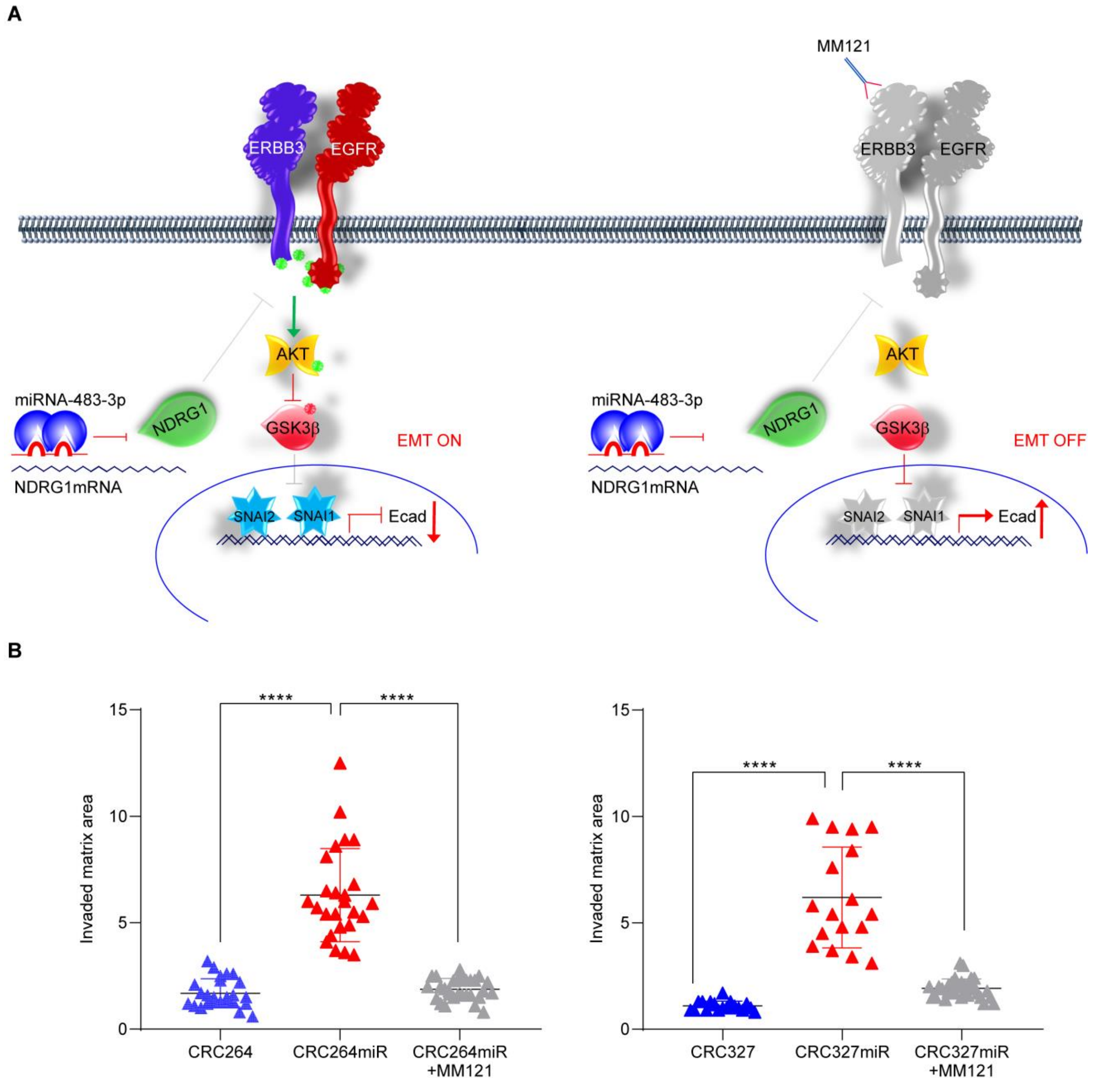

Fig. S4. Selective ERBB3 inhibition dampens miRNA-483-3p-induced invasive growth. **A** Schematic representing the MM121 mechanism of action and its outcomes on ERBB3/EGFR downstream signaling and EMT process. **B** Morphometric analyses of areas invaded by spheroids in 3D assays shown in Fig. 4B (n = 3). Box plots represent fold change of areas invaded by each spheroid (dots) at day 4 vs day 0 (n=3). Left: CRC264miR vs CRC264: ***, P=0.0006; CRC264miR + MM121 vs. CRC264miR: ****, P<0.0001. Right: CRC327miR vs. CRC327: ****, P<0.0001; CRC327miR + MM121 vs. CRC327miR: ****, P<0.0001; ANOVA Bonferroni Multicomparison test.

Fig. S5.

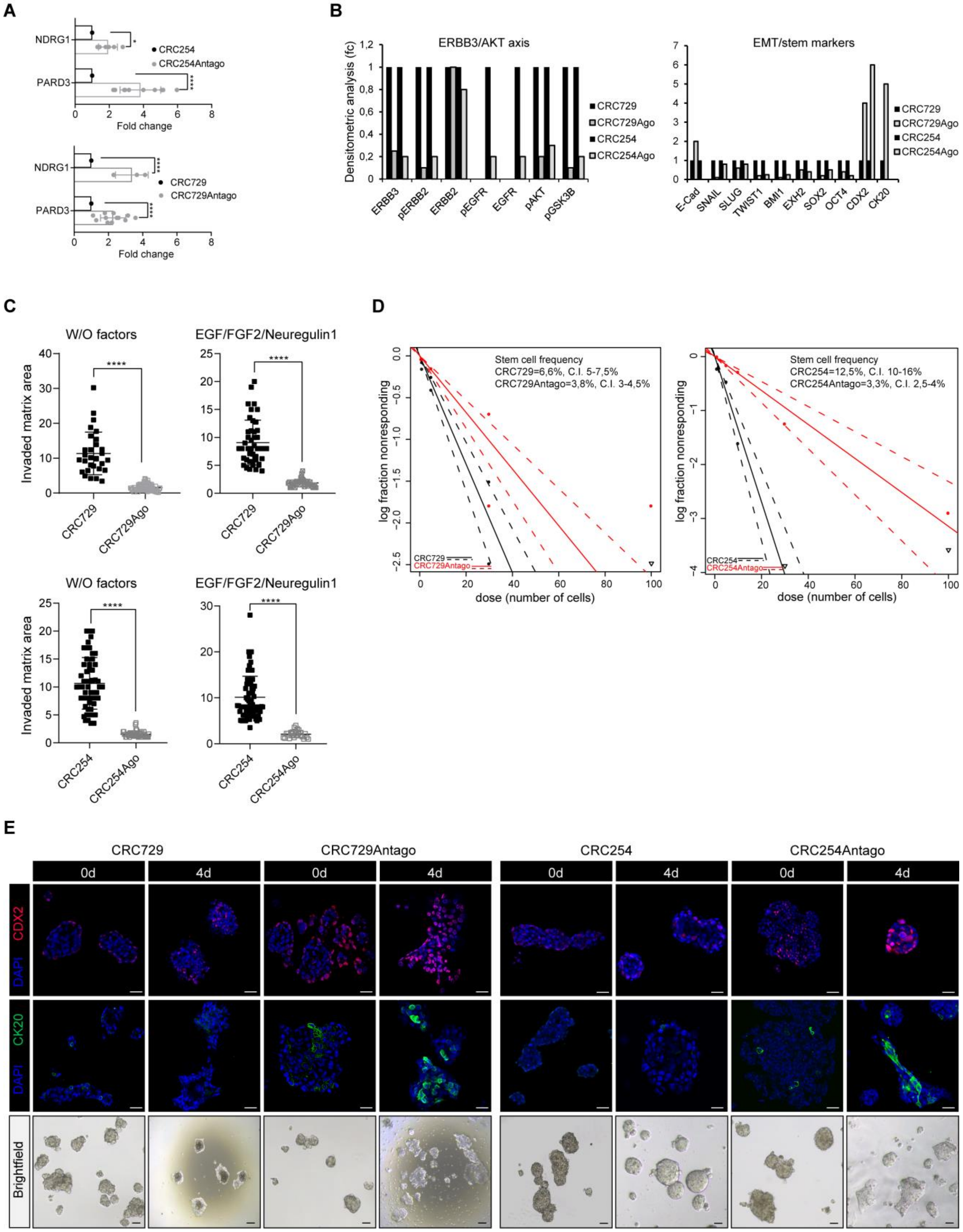
A
NDRG1
PARD3
CRC254
CRC254Antago
Fold change
CRC729
CRC729Antago
B
ERBB3/AKT axis
Densitometric analysis (fc)
CRC729
CRC729Ago
CRC254
CRC254Ago
ERBB3
pERBB2
ERBB2
pEGFR
EGFR
pAKT
pGSK3B
EMT/stem markers
E-Cad
SNAIL
SLUG
TWIST1
BMI1
EXH2
SOX2
OCT4
CDX2
CK20
C
W/O factors
EGF/FGF2/Neuregulin1
Invaded matrix area
CRC729
CRC729Ago
CRC254
CRC254Ago
D
Stem cell frequency
CRC729=6,6%, C.I. 5-7,5%
CRC729Antago=3,8%, C.I. 3-4,5%
log fraction nonresponding
dose (number of cells)
CRC254=12,5%, C.I. 10-16%
CRC254Antago=3,3%, C.I. 2,5-4%
E
CRC729
CRC729Antago
CRC254
CRC254Antago
0d
4d
CDX2
DAPI
CK20
Brightfield

Fig. S5. AntagomiRNA-483-3p upregulates NDRG1, impairs ERBB3 activity and inhibits invasive and tumorigenic properties in m-colospheres endogenously overexpressing miRNA-483-3p. **A** *NDRG1* and *PARD3* mRNA expression in parental CRC729 and CRC254, and in CRC729 and CRC254 transduced with AntagomiRNA-483-3p. Upper panel: CRC254 vs CRC254Antago, *NDRG1* *, *P*=0.0371; *PARD3* ****, *P*<0.0001. Lower panel: CRC729 vs CRC729Antago, *NDRG1*****, *PARD3* ****, *P*<0.0001; one-way ANOVA). **B** Densitometric analyses of western blots shown in Fig. 5A (left) and 5F (right) (n = 3). **C** Morphometric analyses of areas invaded by colospheres in 3D assays shown in Fig. 5D (n = 3). Top: CRC729 and CRC729Antago. Bottom: CRC254 and CRC254Antago. Box plots represent fold change of areas invaded by each spheroid (dots) at day 4 vs day 0 (n = 3) (CRC729 vs. CRC729Antago w/o factors: ****, *P*<0.0001; CRC729 vs. CRC729Antago EFN: ****, *P*<0.0001; CRC254 vs. CRC254Antago w/o factors: ****, *P*<0.0001; CRC254 vs. CRC254Antago: ****, *P*<0.0001; ANOVA Bonferroni Multicomparison test). **D** In vitro limiting dilution sphere-forming assay. For each m-colosphere, plots generated by the ELDA software are shown, reporting the estimated stem cell frequency (percentage of clonogenic cells) with confidence intervals (C.I.) Left: CRC729 and CRC729Antago. Right: CRC254 and CRC254Antago. **E** Differentiation assay. M-colospheres were cultured for 4 days in pro-differentiating conditions (adhesive substrate and basal medium containing 10% FBS). Immunofluorescent stainings for CDX2 and CK20 and bright field acquisition are shown. Nuclei were counterstained with DAPI. Scale bar: 50 μm (n=3).

2. Supplementary Tables

Table S1. List of probes, primers and lentiviral constructs.

**Probe**	**Source**	**Identifier**
TaqMan® miRNA assay	Thermo Fisher Scientific	hsa-miR-483-3p
TaqMan® miRNA assay RNU48	Thermo Fisher Scientific	ID001006
IGF2IN	Thermo Fisher Scientific	Hs01005963_m1
SNAI1	Thermo Fisher Scientific	Hs00195591_m1
NANOG	Thermo Fisher Scientific	Hs02387400_g1
EZH2	Thermo Fisher Scientific	Hs01016789_m1
BMI1	Thermo Fisher Scientific	Hs00995536_m1
EGFR	Thermo Fisher Scientific	Hs01076078_m1
ERBB2	Thermo Fisher Scientific	Hs01001580_m1
ERBB3	Thermo Fisher Scientific	Hs00176538_m1
NDRG1	Thermo Fisher Scientific	Hs00608387_m1
NDRG1	Themo Fisher Scientific	Hs00608388_m1
NDRG1	Themo Fisher Scientific	Hs00608389_m1
UBC	Thermo Fisher Scientific	Hs00824723_m1
GADPH	Thermo Fisher Scientific	Hs99999905_m1
TBP	Thermo Fisher Scientific	Hs00427621_m1
B2M	Thermo Fisher Scientific	Hs00187842_m1
ACTB	Themo Fisher Scientific	Hs99999903_m1
**Primers**		**5'-3' SEQUENCE**
CDH1-fw	SIGMA-ALDRICH	TTGAAAGAGAAACAGGATGGCT
CDH1-rev	SIGMA-ALDRICH	TCATTCTGATCGGTTACCGTGA
NDRG1-fw	SIGMA-ALDRICH	GACATGCAGGCACCTCTTTT
NDRG1-rev	SIGMA-ALDRICH	CCTGCTTTTGCTGCACATTA
PARD3-fw	SIGMA-ALDRICH	CATACGCTTGTTCGGTCCTG
PARD3	SIGMA-ALDRICH	TCATGATAGACACAGGGCCA
GAPDH-fw	SIGMA-ALDRICH	CCCTTCATTGACCTCAACTACA
GAPDH -rev	SIGMA-ALDRICH	ATGACAAGCTTCCCGTTCTC
TBP-fw	SIGMA-ALDRICH	CTTCGGAGAGTTCTGGGATTG
TBP-rev	SIGMA-ALDRICH	CACGAAGTGCAATGGTCTTTAG
**Lentiviral constructs and plasmids**		
Lentivector-based MicroRNA Precursor construct	System Biosciences	PMIRH483-3P PA/AA1
miRZIP Lentiviral-based anti-microRNAs	System Biosciences	MZIP483-3P PA/AA1
pLV[shRNA]-EGFP>Scramble_shRNA	Vector Builder	VB010000-0009mxc
pLV[shRNA]-EGFP>hNDRG1[shRNA#3]	Vector Builder	VB900080-6671zte
pLV[shRNA]-EGFP>hNDRG1[shRNA#4]	Vector Builder	VB210902-1282umu
pLV[shRNA]-EGFP>hNDRG1[shRNA#5]	Vector Builder	VB210902-1283vpc
miTarget™ miRNA 3' UTR NDRG1 WT	GeneCopoeia	HmiT120908-MT06
miTarget™ miRNA 3' UTR NDRG1 Mut	GeneCopoeia	CS-HmiT120908-MT06-01
pEZX-MT06-Luc empty vector	GeneCopoeia	CmiT000001-MT06

Table S2. List of antibodies.

Antibody (cat Number)	Concentrations	Company	RRID
AKT (9272)	1:1000 WB	Cell Signaling Tech.	AB_329827
Actin beta (D6A8)	1:10000 WB	Cell Signaling Tech.	AB_2797972
BMI1, clone D20B, (6964)	1:100 IF; 1:1000 WB	Cell Signaling Tech.	AB_10828713
Caspase-3 (9662S)	1:1000 WB	Cell Signaling Tech.	AB_331439
CDX2 (32977S)	1:100 IF; 1:1000 WB	Cell Signaling Tech.	AB_2077043
EGF Receptor, clone D38B1(4267)	1:100 IF; 1:1000 WB	Cell Signaling Tech.	AB_2246311
EZH2 (5246)	1:100 IF; 1:1000 WB	Cell Signaling Tech.	AB_10694683
HER3, clone1B2 (4754)	1:1000 WB	Cell Signaling Tech.	AB_10691324
NANOG (4903)	1:1000 WB	Cell Signaling Tech.	AB_10559205
p-AKT Ser473, clone 736E16 (3787)	1:1000 WB	Cell Signaling Tech.	AB_331170
p-EGFR Y1068, clone 15A2 (2234)	1:100 IF; 1:1000 WB	Cell Signaling Tech.	AB_331701
p-GSK-3β (Ser9) (D85E12)	1:1000 WB	Cell Signaling Tech.	AB_2798445
p-HER3 Y1289 (4791)	1:1000 WB	Cell Signaling Tech.	AB_2099709
p-p38 T180/Y182 (9215)	1:1000 WB	Cell Signaling Tech.	AB_331762
pHER2 Y1248 (2247)	1:1000 WB	Cell Signaling Tech.	AB_331725
pMEK1/2 S217/221 (9154)	1:1000 WB	Cell Signaling Tech.	AB_2138017
YAP/TAZ, clone D24E4 (8418)	1:1000 WB; 1:50 IHC	Cell Signaling Tech.	AB_10950494
E-Cadherin (610182)	1:1000 WB; 1:100 IF; 1:100 IHC	BD	AB_397581
MET (C-12)	1:1000 WB	Santa Cruz	AB_631940
SNAI 1 (H130)	1:1000 WB	Santa Cruz	AB_2239533
SLUG, clone A-7 (sc-166476)	1:1000 WB; 1:50 IHC	Santa Cruz Biotechnology	AB_2191897
ERBB3, (05-390)	1:100 IF	Millipore	AB_11211839
OCT4, clone 10H11.2 (MAB4401)	1:1000 WB	Millipore	AB_2167852
Vimentin (CBl202)	1:100 IF; 1:1000 WB	Millipore	AB_93387
Twist1 (T6451)	1:1000 WB	SIGMA	AB_609890
ZEB1 (HPA027524)	1:1000 WB; 1:1000 IHC	SIGMA	AB_1844977
NDRG1 (AB-80570)	1:1000 WB; 1:50 IHC	Immunological sci.	AB_2892071
pIGF1R Y1161 (ABP-0367)	1:1000 WB	Immunological sci.	AB_2861138
pERK1/2 T202/Y204 (MAB94122)	1:1000 WB	Immunological sci.	AB_2858271
CDX2 (IR080)	ready to use, IHC	Agilent	AB_2858275
Cytokeratin 20	1:1000 WB	Abcam	AB_1310117
Alexa Fluor 488 donkey anti rabbit Ig H+L) (A21206)	1:750 IF	Jackson Lab	AB_2535792
Alexa Fluor 555 donkey anti mouse Ig (H+L) (A31570)	1:750 IF	Jackson Lab	AB_2535853
Peroxidase-conjgated AffiPure Goat Anti-Mouse IgG (H L) (115-035-003)	1:20000 WB	Jackson Lab	AB_10015289
Peroxidase-conjgated AffiPure Goat Anti-Rabbit IgG (H L) (111-035-003)	1:20000 WB	Jackson Lab	AB_2313567

Table S4. Clinical and molecular data of metastatic colorectal cancer patients and m-colospheres.

Case ID	*Sex*	*Age*	*OS*	*Tumor stage and site*	*Genetic status*	*IGF2 expression**	*miR483-3p expr.#*	*M-colosphere derivation*	*M-colosphere availability*
CRC0030	M	68	36	IV, distal sigmoid	Quadruple WT	high	high	no	no
CRC0069	M	69	23	IV, rectal	Quadruple WT	high	high	no	no
CRC0081	M	61	64	IV, sigmoid	Quadruple WT	high	high	yes	yes
CRC0177	F	59	88	IV, sigmoid	Quadruple WT	high	high	yes	yes
CRC0202	M	77	37	III, rectal	Quadruple WT	high	high	yes	yes
CRC0254	F	47	58	IV, sigmoid	Quadruple WT	high	high	yes	yes
CRC0285	M	63	49	III, rectal-sigmoid	Quadruple WT	high	high	yes	yes
CRC0330	F	51	54	III, sigmoid	KRAS G13D	high	high	no	no
CRC0403	n.a	n.a	n.a	n.a	Quadruple WT	high	high	yes	yes
CRC0442	F	49	25	IV, rectal	Quadruple WT	high	high	yes	yes
CRC0544	M	75	41	III,colon	KRAS G12D	high	high	yes	yes
CRC0705	M	51	n.a	IV, rectal	KRAS G12V	high	high	yes	yes
CRC0729	F	n.a.	48	n.a.	Quadruple WT	high	high	yes	yes
CRC1063	47	F	17	IV ,colon	BRAF K601E	high	high	yes	yes
CRC1083	68	F	30	n.acolon	KRAS G13D	high	high	yes	yes
CRC1090	F	70	n.a	n.a colon.	KRAS G12V, PI3CA Q546K	high	high	no	no
CRC1449	M	60	n.a	n.a rectal	Quadruple WT	high	high	no	no
CRC0059	F	81	29	II, splenic flexure	Quadruple WT	low	low	no	no
CRC0078	M	54	57	IV, rectal-sigmoid	Quadruple WT	low	low	yes	yes
CRC0104	F	64	34	IV,colon	PI3CA H1047R	low	low	yes	yes
CRC0109	M	73	90	IV, rectal	Quadruple WT	low	low	yes	no
CRC0125	F	51	18	IV, sigmoid	Quadruple WT	low	low	yes	no
CRC0136	F	50	79	IV, colon	Quadruple WT	low	low	yes	no
CRC0147	F	55	90	IV, sigmoid	Quadruple WT	low	low	yes	no
CRC0148	M	75	95	III, sigmoid	KRAS G12D	low	low	no	no
CRC0151	F	59	36	III B, colon	Quadruple WT	low	low	yes	yes
CRC0152	M	59	79	IVA, lower rectal	Quadruple WT	low	low	yes	no
CRC0155	M	70	41	IV, rectal	KRAS G12D	low	low	no	no
CRC0167	M	74	86	IV, colon	KRAS G12C	low	low	yes	yes
CRC0179	M	70	48	IV, colon+sigmoid	Quadruple WT	low	low	yes	no
CRC0199	F	65	16	IV, cecum	Quadruple WT	low	low	no	no
CRC0252	M	63	106	IIA, colon	Quadruple WT	low	low	no	no
CRC0261	M	55	24	IVA, rectal	KRAS G12V	low	low	yes	yes
CRC0262	M	63	42	IV, rectal	Quadruple WT	low	low	yes	no
CRC0264	M	69	72	IV, splenic flexure	Quadruple WT	low	low	yes	yes
CRC0291	M	71	22	IV, colon	KRAS G12D	low	low	yes	yes
CRC0316	F	66	25	IV, rectal	Quadruple WT	low	low	no	no
CRC0322	n.a	n.a	n.a	n.a	Quadruple WT	low	low	yes	yes
CRC0327	M	57	145	IV, sigmoid	Quadruple WT	low	low	yes	yes
CRC0394	M	72	48	IV,rectal	Quadruple WT	low	low	yes	yes
CRC0480	F	68	72	III B, sigmoid	BRAF V600E	low	low	yes	yes
CRC0574	M	66	83	IV, colon	Quadruple WT	low	low	yes	yes
CRC0578	F	48	n.a	n.a, colon	Quadruple WT	low	low	yes	yes
CRC0687	F	49	44	IV,cecum	KRAS G13D	low	low	yes	yes
CRC0721	F	61	n.a	n.a, colon	KRAS Q61H	low	low	yes	yes

CRC080	M	53	103	IV, sigmoid	Quadruple WT	low	low	no	no
CRC1078	F	61	59	III, rectal	KRAS G12D	low	low	yes	yes
CRC1139	F	58	16	IV, sigmoid, splenic flexure	KRAS G12D	low	low	yes	yes
CRC1145	F	n.a.	39	n.a.	Quadruple WT	low	low	yes	no
CRC1157	M	n.a.	n.a.	n.a.	Quadruple WT	low	low	yes	no
CRC1182	F	54	41	IV, colon	KRAS G12A	low	low	yes	yes
CRC1257	M	62	15	IV, sigmoid	KRAS G12A	low	low	yes	yes
CRC1313	M	65	12	IV, sigmoid	Quadruple WT	low	low	yes	no
CRC1331	F	57	32	IV, splenic flexure	Quadruple WT	low	low	yes	no
CRC1342	M	47	48	IV, rectal	Quadruple WT	low	low	yes	no
CRC1430	F	60	58	IIIB rectal sigmoid junction	Quadruple WT	low	low	yes	yes
CRC1448	M	40	17	IV, rectal	KRAS A146P	low	low	yes	yes

OS: overall survival (months)
* As shown by Zanella et al., 2015
# As shown in Fig. S1B
n.a.: not available
Red: cases with high IGF2 expression and available m-colospheres
Blue: cases with low IGF2 expression and available m-colospheres

Table S5. Expression of miRNA-483-3p and IGF2 gene across 39 colorectal cancer celllines from the Cancer Cell Line (CCL) Encyclopedia dataset.

miR-483-3p_exp	CCL_name	log10_miR-483-3p	log10_IGF2	log_10 NDRG1
high	SNU1033	2.242615958	3.684616036	1,662084453
high	C2BBE1	2.093876755	2.935909051	1,579718712
high	GP2D	2.233757363	2.871763419	1,001549363
high	LS1034	2.731306664	3.748422653	1,149757041
high	CL14	2.648252644	4.16025319	1,614654001
low	SW1116	1.36660971	0.299761227	1,7313715
low	SNUC2A	0.85672889	0.509278832	1,541971256
low	LS123	1.080626487	0.731098944	1,977677551
low	SNU1197	1.36567514	0.446535778	1,976420608
low	CW2	1.079543007	0.736977863	2,15497368
low	HT115	1.079181246	0.141759872	2,012895963
low	SNUC5	1.365862215	0.281328711	1,603149112
low	SNUC4	1.104828404	0.180335013	1,340978449
low	HCT15	1.382917135	0.187984223	1,534461874
low	SW1463	1.068185862	0.336489855	1,821987576
low	CCK81	1.439332694	0.570933516	1,614359121
low	LS513	1.261738547	0.249424409	1,747713163
low	SW837	1.42258984	0.810526077	1,457982591
low	HT55	0.933993164	0.45720862	1,703177439
low	MDST8	1.197280558	0.619699197	2,230362845
low	SNU81	1.286007122	0.282378684	2,38985357
low	SNU1040	1.148602655	0.660534771	2,260822374
low	HCC56	1.09482038	0.356822978	1,278629521
low	RCM1	1.137037455	0.465385852	2,358445336
low	RKO	0.965201701	0.149338799	1,166501789
low	HCT116	1.204391332	1.22245863	2,014128552
low	LS411N	0.873320602	0.071598687	1,63459177
low	SW48	1.214313897	0.460131583	2,110587472
low	LS180	1.192009593	0.196073526	1,604107444
low	SW403	1.068185862	0.292755089	2,004517531
low	NCIH747	1.067814511	0.685512057	1,366484825
low	SW1417	1.083144143	0.444752983	2,205547513
low	SW948	1.200029267	0.267994748	2,825992544
low	HT29	1.059563418	1.72286851	1,842418095
low	KM12	1.080626487	0.073065613	1,066054072
low	CL40	1.230959556	0.521262389	2,123476143
low	COLO320	1.020361283	0.465709773	0,747124188
low	CL11	1.100715087	0.420512363	2,342333884
low	CL34	1.036628895	0.336137475	2,090704528

3. Description of Table S3 (separate .xlsx file).

Differentially expressed genes in CRC264, CRC264miR, CRC327 and CRC327miR; ensID (Ensembl ID), symbol (gene symbol), Log2FC (Fold Change), pvalue, FDR are shown.

Sheet 1: CRC2327 vs CRC327miR.

Sheet 2: CRC264 vs CRC264miR.
